# A massively parallel reporter assay reveals focused and broadly encoded RNA localization signals in neurons

**DOI:** 10.1093/nar/gkac806

**Published:** 2022-09-26

**Authors:** Martin Mikl, Davide Eletto, Malak Nijim, Minkyoung Lee, Atefeh Lafzi, Farah Mhamedi, Orit David, Simona Baghai Sain, Kristina Handler, Andreas E Moor

**Affiliations:** Department of Biosystems Science and Engineering, ETH Zürich, Basel, Switzerland; Department of Human Biology, University of Haifa, Haifa, Israel; Department of Biosystems Science and Engineering, ETH Zürich, Basel, Switzerland; Department of Human Biology, University of Haifa, Haifa, Israel; Department of Biosystems Science and Engineering, ETH Zürich, Basel, Switzerland; Department of Biosystems Science and Engineering, ETH Zürich, Basel, Switzerland; Department of Biosystems Science and Engineering, ETH Zürich, Basel, Switzerland; Department of Human Biology, University of Haifa, Haifa, Israel; Department of Biosystems Science and Engineering, ETH Zürich, Basel, Switzerland; Department of Biosystems Science and Engineering, ETH Zürich, Basel, Switzerland; Department of Biosystems Science and Engineering, ETH Zürich, Basel, Switzerland

## Abstract

Asymmetric subcellular mRNA localization allows spatial regulation of gene expression and functional compartmentalization. In neurons, localization of specific mRNAs to neurites is essential for cellular functioning. However, it is largely unknown how transcript sorting works in a sequence-specific manner. Here, we combined subcellular transcriptomics and massively parallel reporter assays and tested ∼50 000 sequences for their ability to localize to neurites. Mapping the localization potential of >300 genes revealed two ways neurite targeting can be achieved: focused localization motifs and broadly encoded localization potential. We characterized the interplay between RNA stability and localization and identified motifs able to bias localization towards neurite or soma as well as the trans-acting factors required for their action. Based on our data, we devised machine learning models that were able to predict the localization behavior of novel reporter sequences. Testing this predictor on native mRNA sequencing data showed good agreement between predicted and observed localization potential, suggesting that the rules uncovered by our MPRA also apply to the localization of native full-length transcripts.

## INTRODUCTION

The cytoplasm is a tightly regulated space that accommodates countless parallel tasks in specialized compartments. Asymmetric subcellular mRNA distributions have been observed in a variety of polar cell types (1–4). mRNA localization might be an energy-efficient way to generate corresponding protein gradients, it might prevent harmful protein effects by ectopic activity or accumulation, and it could accelerate cellular response to extrinsic stimuli by activation of localized protein translation (5).

Neurons show a high degree of functional compartmentalization, which to a large part is achieved through transporting specific transcripts into dendrites or axons, where they are available for local (and sometimes activity-dependent) translation (6). RNA localization in neurons was first demonstrated using *in situ* hybridization techniques (7,8) and also revealed differences in the localization characteristics between neuronal cell types and brain regions (9). Fractionation-based approaches allowed for the characterization of the entire pool of dendritically localized mRNAs (10,11), revealing the richness of the local transcriptome and subsequently proteome (12,13) as well as isoform-specific regulation (14). Recent advances in spatial transcriptomics have enabled the study of dendritically localized RNAs at single cell resolution (15), with massively parallel hybridization approaches (16), and with expansion sequencing (17). This body of work reveals a wealth of mRNA localization patterns. The extent of this phenomenon in steady-state and its dynamic adaptation in physiology (18) suggest that RNA localization is a tightly regulated process.

Most studied RNA localization phenotypes depend on interactions between RNA binding proteins (RBP) and the 3′ untranslated region (3′UTR) of a gene (19,20). Approximately 1500 RBPs, interacting with a variety of RNA molecules, have been identified in mammalian cells (21). Several well-characterized localized transcripts are linked by RBPs to molecular motors, which enable active transport on the cytoskeleton (22). Alternatively, localized RBPs have been shown to prevent RNA degradation (23) or to capture and anchor transcripts and thereby create subcellular asymmetry in RNA localization (19). The localization of beta-actin mRNA to the leading edge in fibroblasts and to axonal growth cones in neurons are well-studied examples of RNA localization (24): they have been found to be mediated by the RBP ZBP1 binding to a ‘zip-code’ in the three prime untranslated region (3′UTR) of the beta-actin mRNA (25). Apart from a very limited number of such cases with a transparent link between a specific motif and a trans-acting factor mediating RNA sorting, the link between sequence and localization potential remains obscure and we still lack a systematic understanding of how the transcript sorting machinery works in a sequence-specific manner.

Large-scale testing of rationally designed or random sequence libraries has immensely contributed to elucidating the regulatory grammar of transcription (26–30)^,^ splicing (31–35), polyadenylation (36,37), miRNA-mediated regulation (38), other forms of translational control (39,40), and RNA nuclear enrichment and export (41–43) demonstrating the power and universal applicability of such approaches. Although they have become an experimental pillar of studies in gene regulation, a dedicated high-throughput systematic attempt to dissect the regulatory logic of subcellular RNA localization is still lacking.

Here, we address our gaps in understanding the regulatory code of mRNA localization in neurons by combining subcellular transcriptomics and massively parallel reporter assays. This enabled us to functionally test endogenous and synthetic localization elements with unprecedented scale, describe ways how the localization potential can be encoded in the 3′UTR sequence, identify potential protein mediators of localization, and train computational models to attempt prediction of the localization of novel sequences.

## MATERIALS AND METHODS

### Synthetic library design

Oligonucleotides were designed to maintain a constant length of 198 nt. Restriction sites used for cloning were excluded from the design. All the variants were composed of an 18 nt forward primer, 12 nt barcode sequence, 150 nt variable region and 18 nt reverse primer sequences. DNA barcodes were designed to differ from any other barcode in the library in at least 3 nt.

Design of the subsets of the library was carried out in Python; the scripts are available at github.com/martinmikl/RNAloc_MPRA.

Tiling of endogenous 3′UTRs: Tiles of 150 nt length were chosen, starting from the first position after the stop codon and extending until the most distal poly-adenylation site, excluding tiles that would contain a poly-adenylation site themselves to avoid cleavage within the 3′UTR reporter construct. Genes were selected based on previous transcriptomics data obtained from cultured neurons (13,14,44,45). These consisted of the following groups of genes (see also Supplementary Table S1): 14 RNAs identified in multiple prior studies (e.g. Camk2a, Map2, Shank1), yielding 715 tiles; the 40 RNAs found most often in single dendrites analyzed by Middleton *et al.* (45), yielding 1178 tiles; 347 dendrite enriched genes found by Middleton *et al.*, yielding 7948 tiles; 127 neurite enriched (logFC > 2, adj. *P*-value < 0.01) genes identified by (13), yielding 2355 tiles; 33 genes with evidence from two studies (14,45) for differential localization behavior of 3′UTR isoforms, yielding 1203 tiles; 5 genes (Rragb, St6gal1, Gpr17, Ogt, Pgap1) found enriched in the soma fraction in all of the previous studies (13,14,44,45), yielding 710 tiles. After removing duplicate sequences and sequences containing potential poly-adenylation sites, the final library covering endogenous 3′UTRs consisted of 13 754 tiles. Four out of the original 315 genes were not used for the tiling due to the short length of their 3′UTR and the presence of potential poly-adenylation sites.

Multiple barcode controls: We added multiple variants to the library that contained the same variable region, but different barcodes, in order to gauge potential effects of the barcode and the technical noise of our assay. The final library contained 57 sequences with 3 barcodes, 45 sequences with 4 barcodes, 21 sequences with 6 barcodes, 18 sequences with 5 barcodes and 1 sequence with 7 barcodes.

RBP motif insertions and deletions: We assembled a list of potential RBP binding sites consisting of the results of our own bioinformatic analysis (see details below: RBP motif enrichment analyses) and previously reported motifs (neurite-enriched according to Middleton *et al.*: ATCAACG, ATCATCG, TTCGAT, CCGCAA, GTGGGT; neurite-enriched according to Taliaferro *et al.*: GCTGCT, CTGCTG, GCGCTG, CTGGAC, CCTGCT, TCTGGA, CCCCAA, CTGCCC, ACACTG, TTTTCA, TTTTTT, ATACAG; soma-enriched according to Taliaferro *et al.*: TAGGTC, TCTTCT, CTCTTT, TCTCTT, TCTCTC, AGGTAA).

Motif mutations: We mutated 44 neurite- and 84 soma-enriched motifs in endogenous 3′UTR tiles (see above) in the following way: for genes with evidence for neurite enrichments (all groups of genes mentioned above with the exception of the five genes found enriched in the soma compartment in all of the previous studies, we scanned each tile for the presence of any of the 44 neurite-enriched motifs. If a motif was present, we included a sequence in the library which corresponded to the endogenous 3′UTR tile, but had all instances of this motif mutated (replaced by a random sequence). For genes previously found enriched in the soma compartment and for those genes with differential localization behavior of 3′UTR isoforms we scanned each tile for the presence of any of the 84 soma-enriched motifs. If a motif was present, we included a sequence in the library which corresponded to the endogenous 3′UTR tile, but had all instances of this motif mutated (replaced by a random sequence).

Motif insertions: We introduced these motifs in different configurations:

We inserted two copies of 21 neurite- and 15 soma-enriched motifs in 189 native contexts (150 nt tiles from endogenous 3′UTRs) at positions 50 and 100 in order to test the activity of the motifs in as many native contexts as possible.

We inserted one, two, three or four copies of 21 neurite- and 15 soma-enriched motifs in 22 native contexts (150 nt tiles from endogenous 3′UTRs) at positions 30, 60, 90 and 120 in order to determine if the effect of the motif increases with the number of times it is present.

We inserted one or two copies of 44 neurite-enriched motifs in 22 native contexts (150 nt tiles from endogenous 3′UTRs) at position 50 or 50 and 100, in the native sequence or embedded within an artificial 9 bp hairpin structure to determine the effect of the local secondary structure on motif effects.

We inserted 66 synthetic sequences (see below) in 69 native contexts (150 nt tiles from endogenous 3′UTRs) at position 30.

### Synthetic library cloning

The cloning steps were performed essentially as described previously (32,37). We obtained the oligonucleotide library from Twist Bioscience as a pool. The two subsets of this pool corresponding to native 3′UTR tiles and designed sequence alterations (mutations and motif insertions) were defined by unique amplification primers. The oligo pool was resuspended in 10 mM Tris buffer, pH 8.0 to a concentration of 20 ng/μl. We amplified both libraries by performing four PCR reactions, each of which contained 19 μl of water, 1 μl of the oligo pool, 10 μl of 5× Herculase II reaction buffer, 5 μl of 2.5 mM deoxynucleotide triphosphate (dNTPs) each, 5 μl of 10 μM forward primer, 5 μl of 10 μM reverse primer, and 1 μl Herculase II fusion DNA polymerase (Agilent Technologies). The parameters for PCR were 95°C for 1 min, 14 cycles of 95°C for 20 s, and 68°C for 1 min, each, and finally one cycle of 68°C for 4 min. The oligonucleotides were amplified using library-specific common primers, which have 18-nt complementary sequence to the single-stranded 198-mers and a tail containing SgsI (forward primer) and SdaI (reverse primer) restriction sites (native 3′UTR tiles library: cacaGGCGCGCCaCGAAATGGGCCGCATTGC and cacaCCTGCAGGaTCGTCATCAGCCGCAGTG; designed sequence alterations library: cacaGGCGCGCCaGACAGATGCGCCGTGGAT and cacaCCTGCAGGaGCATTGGATCGGGTGGCT. The PCR products were concentrated using Amicon Ultra, 0.5 ml 30K centrifugal filters (Merck Millipore). The concentrated DNA was then purified using PCR mini-elute purification kit (Qiagen) according to the manufacturer's protocol. Purified library DNA was cut with the unique restriction enzymes SgsI and SdaI (Fermentas FastDigest) for 2 h at 37°C in two 40-μl reactions containing 4 μl fast digest (FD) buffer, 1 μl SgsI enzyme, 1 μl SdaI enzyme, 18 μl DNA and 16 μl water, followed by heat inactivation for 20 min at 65°C. Digested DNA was purified, first using PCR mini-elute purification kit (Qiagen) and then using 2.2x SPRI beads (Beckman-Coulter).

The master plasmid for inserting the library was created by introducing a synthetic sequence containing a stop codon, a primer binding site and restriction sites for SgsI and SdaI into the Bsp1704I site at the 3′ end of the GFP coding region of pcDNA3-EGFP (Addgene #13013). The modified plasmid was cut with SgsI and SdaI (Fermentas FastDigest) in a reaction mixture containing 6 μl FD buffer, 3 μl of each enzyme and 3.5 μg of the plasmid in a total volume of 60 μl. After incubation for 2.5 h at 37°C, 3 μl FD buffer, 3 μl alkaline phosphatase (Fermentas) and 24 μl water were added and the reactions were incubated for an additional 30 mins at 37°C followed by 20 min at 65°C. Digested DNA was purified using a PCR purification kit (Qiagen). The digested plasmid and DNA library were ligated for 30 min at room temperature in 15 μl reactions, containing 150 ng plasmid and the insert in a molar ratio of 1:1, 1.5 μl FastLink 10 × ligation buffer, 1.5 μl ATP and 1 μl FastLink DNA ligase (Lucigen Corporation), followed by heat inactivation for 15 min at 70°C. Ligated DNA was transformed into *E. cloni* 10G SUPREME Electrocompetent Cells (Lucigen) (2 μl of the ligation mix per reaction) using Biorad GenePulser Xcell (Voltage 1800V, capacitance 25 uF, resistance 200 Ohm, 1 mm cuvettes), which were then plated on 4 Luria broth (LB) agar (200 mg/ml amp) 15-cm plates per transformation reaction (25 μl). The rationally designed (12,809 variants) and the native (5000 variants) parts of the library were cloned separately. For the two libraries we collected around 1.2 × 10^6^ and 2.3 × 10^6^ colonies, respectively, the day after transformation by scraping the plates into LB medium. Library-pooled plasmids were purified using a NucleoBond Xtra EndoFree midi prep kit (Macherey Nagel). To ensure that the collected plasmids contain only a single insert of the right size, we performed colony PCR (at least 30 random colonies per library).

For cloning of the barcode-only library used for assessing the effect of the barcode sequence, we obtained pairs of oligonucleotides containing the same constant sequences and overhangs for cloning as the full library sequences and a randomized 12mer. These oligonucleotides were annealed and cloned into the master plasmid as described above. The MPRA was carried out for both libraries (the full sequence as well as the barcode-only library) in an identical way as described below.

### Cell culture

CAD cells were acquired from Sigma-Aldrich (#ATCC^®^ CRL-11179); Neuro-2a cells were a gift from Prof. Peter Scheiffele, U. Basel, Switzerland. CAD cells were grown in DMEM/F-12 (Gibco) supplemented with 8% fetal bovine serum (Gibco, #10270–106) and 1% penicillin–streptomycin solution (Gibco, #15140–22). Neuro-2a cells were grown in DMEM (Gibco) supplemented with 10% fetal bovine serum and 1% Penicillin-Streptomycin solution. The cells were grown in a humidified incubator at 37°C and 5% CO_2_ and split by dissociation by pipetting (CAD cells) or by trypsinizing (TrypLE, ThermoFisher). To induce differentiation into a more neuron-like phenotype, medium was changed to differentiation medium (DMEM/F-12 with 0.8% fetal bovine serum and 1% penicillin–streptomycin solution and DMEM with 1% fetal bovine serum and 1% penicillin–streptomycin solution, respectively).

### Library transfection and neurite- and soma-specific RNA extraction

For quantifying the abundance of library sequences in neurite and soma, CAD and Neuro-2a cells were grown on Millicell Hanging Cell Culture Inserts with a pore size of 3 μm (Millipore #MCSP06H48), adapting experimental pipelines described earlier (13,44,46). Prior to seeding the cells, the bottom of the insert was coated with 100 ul Matrigel (Corning #356237, diluted to 3 mg/ml with PBS). Matrigel was allowed to solidify for 3 h by incubating the plates with the coated inserts bottom up at 37°C and 5% CO_2_. 5 × 10^5^ cells were seeded on each filter in differentiation medium, adding medium also to the bottom compartment of the well. After 24 h, reporter libraries were transfected into the cells using 2.5 ug reporter DNA per well and Lipofectamine 3000 (ThermoFisher) according to the manufacturer's protocol. For library transfections, three biological replicates were performed. Each replicate was pooled from three 6-well plate inserts with transfected cells. Twenty 4 h after transfection soma and neurites were harvested as follows: Wash cells twice with PBS. Aspirate and add as much fresh PBS to wells with inserts as needed so that the volume above the filter insert will remain approximately 1 ml. Scrape off cells growing on top of the insert, wash off cell bodies with P1000 pipette and transfer them to a microcentrifuge tube. Spin down at 300 g for 5 min, take off supernatant and add 300 ul TRIreagent. While spinning down the cell bodies, wash the remaining inserts with PBS. Clean the insert filter thoroughly, scrape the insert to remove all the remaining cell bodies carefully without disrupting the filter, aspirate pbs. Repeat the step at least one more time and check under the microscope if all the cell bodies have been removed; this step is critical for good separation. Aspirate PBS, take out insert, remove filter from the insert with forceps and transfer it to a microcentrifuge tube with 300 ul TRIreagent.

RNA was isolated using Direct-zol RNA miniprep kit (Zymo research #R2051) according to the manufacturer's protocol.

### Measurements of reporter RNA stability

For quantifying the RNA stability associated with the assayed stretches of endogenous 3′UTR sequences, CAD cells were seeded in 6-well plates in differentiation medium and transfected with the reporter library of wild-type 3′UTR sequences 24 h later. The next day, Actinomycin D was added together with fresh medium at a concentration of 20 ng/ml and cells were collected in duplicates at 0, 4 or 24 h after Actinomycin D treatment for RNA isolation using Direct-zol RNA miniprep kit (Zymo research #R2051).

### cDNA synthesis and library preparation for Illumina sequencing

cDNA was synthesized from up to 10 ug total RNA in a 40 ul reaction using SuperScript IV Reverse Transcriptase (ThermoFisher) according to the manufacturer's protocol, with oligo-dT primers containing 6 nt barcodes, a 15 nt unique molecular identifier (UMI) and a partial Illumina read 1 primer sequence. For library preparation, reporter cDNA was PCR amplified using a reporter specific forward primer and a reverse primer binding the anchor sequence of the oligo-dT primer (corresponding to the Illumina TruSeq Read 1 sequence):

20 ul Kapa hifi ready mix, 1.5 ul 10 uM primer reporter-specific forward primer (adding the Illumina TruSeq Read 2 sequence) and 1.5 ul reverse primer (Illumina TruSeq Read 1), 4 ul cDNA, 13 ul water (two reactions per sample). 98°C 3 min, 15 (soma) or 20 (neurite) cycles: 98°C 20 s, 65°C 15 s, 72°C 20 s; 72°C 1 min. After cleaning up the reaction with 1.8× SPRI beads (Beckman-Coulter) Illumina sequencing adaptors (NEBNext Multiplex Oligos for Illumina, NEB #E7600S) were added in a second PCR reaction: 20 ul Kapa hifi ready mix, 1 ul i7 and 1 ul i5 (from NEB #E7600S), 8 ul first PCR product after SPRI beads, 10 ul water. PCR program: 98°C 3 min, 12 cycles: 98°C 20 s, 65°C 15 s, 72°C 20 s; 72°C 1 min. The reactions were cleaned up with SPRI beads (0.6×) and the size of the product was verified using Tapestation (Agilent High Sensitivity D1000 ScreenTape).

### RNAi experiments

siRNA pools targeting mouse Dazap1, Celf1, Celf6 or Hnrnph2 were obtained as a siGENOME SMARTPool from Dharmacon. siRNA transfections were carried out 48–72 h before library or single reporter transfections using Dharmafect 1 according to the manufacturer's protocol. Target knockdown was verified using qRT-PCR (Supplementary Figure S15). Briefly, CAD cells were lysed and total RNA retrotranscribed with Maxima (ThermoFisher) and oligo dT. Gene expression was assessed by the DeltaDelta-Ct method, using Tbp as internal reference.

For MPRA experiments in combination with RNAi, cell were grown on microporous membranes (as described above) and the library was transfected 48 h after siRNA transfections. For these experiments each well constituted a biological replicate.

### *In vitro* transcription and pull-down experiments

10 ul (∼20–35 ng/ul) of cleaned-up PCR amplicon (primer sequences are provided in the table below, used with screening pooled library) were used as template of the in vitro transcription (HiScribe™ T7 Quick High Yield RNA Synthesis Kit; #E2050S, New England Biolabs), performed at 37°C for 16 h, followed by DNAseI treatment (37°C for 15 min). IVT RNAs were then cleaned-up and concentrated (DNA Clean & Concentrator-5; #D4013, Zymo Research).

3′-Desthiobiotin labeling was carried following the manufacturers’ guidelines of Pierce™ RNA 3′ End Desthiobiotinylation (ThermoFisher, #20163). Briefly, ∼115 pmol of each RNA were first subjected to fast denaturation in the presence of 25% v/v DMSO (85°C for 4') to relax second structures, and subsequently labelled at 16°C for 16 h. RNA binding proteins were isolated by the means of Pierce™ Magnetic RNA-Protein Pull-Down Kit (ThermoFisher, #20164). Briefly, 3′-desthiobiotin labelled RNAs were incubated with magnetic streptaividin-coated beads (50 ul of slurry)/each RNA probe) for 30′ at room temperature, under agitation (600 RPM in a ThermoMixer, Eppendorf). 200 ug of cell lysates (in Pierce IP lysis buffer; #87787, ThermoFisher), derived from fully differentiated CAD or N2a cells, were then incubated with 3′-desthiobiotinilated-RNA/streptavidin beads at 4°C for 1h under agitation (600 RPM). Final elution was performed in 50 ul/pull-down. 20 ul of each eluate was then analyzed by M/S.

Incubation times: 30 min @ RT, 600 RPM agitation for the binding of the labeled RNA to the beads; 1 h @ 4°C, 600 RPM agitation for the RBPs to the RNA and 15 min @ 37°C, 600 RPM agitation for the elution.

**Table:** 

T7IVT-302-F	TAATACGACTCACTATAGGagtggaggttcgcccc
IVT-302-R	aaacgagaaggcgtggcc
T7IVT-2034-F	TAATACGACTCACTATAGGtatttattcaaatagcgtgagg
IVT-2034-R	gcatacacaactattaaaagc
T7IVT-2080-F	TAATACGACTCACTATAGGtctagggaattcctggctc
IVT-2080-R	ccccacattaataagaactaaaaac
T7IVT-2535-F	TAATACGACTCACTATAGGctctactgcacttagactctcc
IVT-2535-R	attatcaataatttgtcagctaagg
T7_LocMotif_For	TAATACGACTCACTATAGGcctccccccccccctgt
LocMotif_Rev	ctgcagcaggcaggggcc

The sequence motifs 302, 2034, 2080 and 2535 (Supplementary Table S14) served as negative controls and were used in equimolar combination (1:1:1:1).

### Mass spectrometry

The pulldown samples were subjected to Trichloroacetic (TCA) precipitation:

20 μl of each sample + 80 μl of H_2_O + 100 μl of 10% TCA (5% TCA end concentration). The resulting protein pellets were washed twice with cold acetone, dried and dissolved as follows: 45 μl of 10 mM Tris/2 mM CaCl_2_, pH 8.2 buffer; 5 μl trypsin (100 ng/μl in 10 mM HCl); 0.3 μl trypsin Tris 1 M, pH 8.2 to adjusted to pH 8. The samples were then processed with microwave-assisted digestion (60°C, 30 min) and dried. The dried digested samples were dissolved in 20 μl ddH_2_O + 0.1% formic acid; transferred to the autosampler vials for liquid chromatography–mass spectrometry analysis (LC–MS/MS);

2 μl were injected on a nanoAcquity UPLC coupled to a Q-Exactive mass spectrometer (Thermo Scientific).

The protein identification and quantification was performed using MaxQuant v1.6.2.3 and the data were searched against the Swissprot mouse database. The mass spectrometry proteomics data have been deposited to the ProteomeXchange Consortium via the PRIDE partner repository (47) with the dataset identifier PXD025492.

### Single molecule fluorescence in situ hybridization

smFISH staining was performed according to a previously published protocol (48) with minor adaptations. Briefly, 50 000 CAD or N2A cells were seeded per well in 24-well plate and grown on poly-d-lysine (Gibco, A3890401) coated coverslips (Thermo Scientific A67761333) with cell differentiation medium (Day 1). One day after transfection (Day 5), cells were flushed with cold PBS and fixed in 4% paraformaldehyde (PFA, Santa Cruz Biotechnology, sc-281692) in PBS for 10 min and subsequently washed two times with cold PBS. Fixed cells were permeabilized with 70% ethanol for at least 1 h or maintained overnight at 4°C (Day 6). The permeabilized samples were washed once with wash buffer A (10% Formamide (Ambion, 9342), 20% Stellaris RNA FISH Wash Buffer A (Biosearch Technologies Cat# SMF-WA1-60) in nuclease-free water (Ambion, AM9932)) for 5 min each at 37°C. Probe libraries were designed using the Stellaris FISH Probe Designer (Biosearch Technologies, Inc., Petaluma, CA, see Supplementary Table S19) and covalently coupled to Cy5 (49).

Hybridization mix (200 nM probes in Stellaris hybridization buffer, Biosearch Technologies Cat# SMF-HB1-10 and 10% Formamide) was added after aspirating the wash buffer A and the sample was incubated upside-down facing the buffer at 37°C in the dark for about 18–24 h (within assembled humidified chamber). Hybridization mix was carefully removed and sample was washed once with wash buffer A at 37°C for 30 min each in the dark. Samples were stained with DAPI (Invitrogen, D1306; 10 μg/ml in wash buffer) for 30 min at 37°C in the dark. DAPI solution was aspirated and samples were washed once with wash buffer B (Biosearch Technologies Cat# SMF-WB1-20) for 5 min. Samples on cover glass were gently mounted upside-down with a small drop of ProLong™ Gold (Invitrogen™ P36930). smFISH imaging was performed on a Leica THUNDER Imager 3D Cell Imaging system. 100× NA = 1.4 oil immersion objective lens was used.

smFISH-based quantifications of neurite localization were carried out as follows: We measured fluorescence intensity of the smFISH signal along the dendrite, starting from the cell body. We then applied background subtraction and normalized the values for expression level (cell-body fluorescence intensity). A measure for neurite localization was obtained by taking the fold change in mean signal in distal parts of a neurite (more than 30 μm from the cell body) over the mean signal in proximal parts of the same neurite (between 5 and 30 μm from the cell body).

smFISH-based quantification of nuclear/cytoplasmic ratio was carried out by manually segmenting nuclear, soma, cytoplasmic, and intercellular regions based on the DAPI and GFP background channels in Fiji (50). The intercellular background signal intensity in the Cy5 channel was then subtracted from both the nuclear and cytoplasmic regions of interest before calculating the nuclear / cytoplasmic ratio for each segmented cell.

### Mapping next generation sequencing reads and computing enrichment scores

Mapping was performed using custom-made Python scripts available at github.com/martinmikl/RNAloc_MPRA. To unambiguously identify the library variant, a unique 12-mer barcode sequence was placed at the 5′ end of each variable region. DNA was sequenced on a NovaSeq 6000 sequencer (SP flow cell, paired end: read 1 30 bp, read 2 84 bp) and demultiplexed using bcl2fastq. We used read 2 to determine for each read its variant barcode and discarded all the reads that could not be assigned to a library variant of origin. Furthermore, we extracted the corresponding UMI from read 1 and used the UMI count per library variant per sample as the starting point for all subsequent analyses.

Enrichment (logFC(neurite/soma) and associated p-value) was calculated using edgeR (51) (RRID:SCR_012802, version 3.28.1; based on fitting a generalized linear model and performing likelihood ratio test to test for enrichment) based on UMI counts of each library sequence in the neurite and soma fraction of three biological replicates. RNA decay rates were calculated the same way using edgeR based on two biological replicates for each time point. Positive log fold changes indicate higher than average stability of a sequence compared to the population of library sequences, negative fold changes indicate lower than average stability of a sequence compared to the population of library sequences.

### RBP motif enrichment analyses

In order to find enriched RBP motifs in published localized RNAs, we downloaded 103 position frequency matrices (PFMs) that correspond to 85 human RBPs from the RNAcompete paper (52). These PFMs (which are of length seven or eight) are generated from the alignment of top 10 7-mers determined using all data (i.e. both setA and setB of RNAcompete pool). Rather than using these top 10 7-mers directly, we generated the top 10 n-mers from the PFMs. In this way, we were able to scan for motifs that are longer than seven. An example is the FXR1 RBP for which the PFM inferred by RNAcompete is of length eight. By using the top 10 8-mers in our motif search, we can represent the binding preferences to all eight positions of this PFM. Next, we have collected a set of dendritically localized RNAs and background RNAs from Middleton et al (45), as well as a second set of RNAs localized in Neurite versus Soma from Zappulo et al.(13) (log FC > 1 for RNAs localized in Neurite and log FC> –2 for RNAs localized in Soma to have a comparable set of RNAs in terms of numbers of RNAs in each set). 3′UTRs of the reported localized RNAs were extracted from Ensemble using biomaRt R package (53) for the RNA ids with corresponding match in the database. All extracted 3′UTR sequences were converted to ‘BStringSet’ using Biostrings R package (2.36.1) for the downstream analysis. To calculate the enrichment of each RBP motif in RNAs reported as localized in Dendrites/Neurites versus Background/Soma in Middleton *et al.* and Zappulo *et al.* correspondingly, we used enrich_motifs function from universalmotif R package which provided the number of motif hits for each of the RBP top 10 *n*-mers as well as the corresponding *P*-value and *q*-value.

To calculate the enrichment of each RBP motifs in tiles that are enriched in neurites and tiles that are enriched in soma compared to all tiles, we used *enrich_motifs* function from universalmotif R package which provided the number of motif hits for each of the RBP top 10 *n*-mers as well as the corresponding *P*-value and *q*-value.

Enriched motifs are selected as the ones having a *P*-value <0.001 in Fisher's Exact Test for Count (enrichment test).

To scan DAZAP1 and SU1 on endogenous 3′UTR sequences and library sequences, using the corresponding PWMs, we used the scan_sequence function from universalmotif R package and FIMO from MEME Suite.

For the correlation analysis, we calculated cumulative binding scores for 218 RBP motifs (RNAcompete) in our library sequences taken from native 3′UTRs and computed the Pearson correlation coefficient (and the associated *P*-value and *q*-value at an FDR of 0.1) between this score and the log FC(neurite/soma) in CAD or Neuro-2a cells or the log FC (4 h/0 h Actinomycin D).

### De-novo motif analysis

In addition to the enrichment of known RBP motifs we scanned published localized RNA 3′UTRs for *de-novo* motifs. To do so, for each set of extracted 3′UTRs from localized RNA in Middleton *et al.* and Zappulo *et al.* we used MEME Suite for the following analysis: (i) MEME de-novo motif discovery analysis (54) to discover novel, ungapped motifs in our localized sets of RNAs. We ran this function in a Differential Enrichment mode by providing the Dendrite/Neurite localized 3′UTRs as primary sequences and Background/Soma RNAs as control sequences. We chose anr (Any Number of Repetition) as site distribution of the function and we looked for the 20 *de-novo* motifs. The rest of the parameters were kept as default. (ii) MAST Motif scanning analysis (55) on the set of motifs we had discovered de-novo from the previous step. We provide the MEME xml output as the motif set for the MAST function and the 3′UTR of the Dendrite/Neurite localized RNAs as the sequence sets to scan for the matches to motifs.

### Prediction of localization

Machine learning procedures were carried out using the python scikit-learn and XGBoost package. Initially, from all duplicated sequences (e.g. barcode control sets), which passed filtering (for building the predictive model we only included library sequences for which we obtained at least 500 reads for all samples (3x soma and 3x neurite) together), a single variant was randomly chosen for all subsequent steps to avoid biases resulting from having duplicated sequences, resulting in altogether 36 731 sequences being used. Ten percent of the variants were put aside and used only for evaluation of models built using the other 90%. We chose Gradient Boosting Decision Trees (XGBoost (56)) as the prediction algorithm because it can capture non-linear interactions between features and has proven to be a powerful approach in predicting the effect of regulatory regions (32,40).

We used two sets of features for our prediction: (i) We computed counts of all possible fourmers in each library sequence (not taking into account the barcode and constant sequences like primer binding sites). (ii) We computed cumulative binding scores of a set of 218 RBPs (RNAcompete (52)) for each library sequence (not taking into account the barcode and constant sequences like primer binding sites).

We trained two models: one predicting neurite enrichment, where the positive class was defined as having a log FC > 0 and an associated *P*-value <0.05 in both CAD and Neuro-2a cells. The second model was trained to predict soma enrichment, where the positive class was defined as having a log FC < 0 and an associated *P*-value <0.05 in both CAD and Neuro-2a cells. For prediction of the localization behavior of unseen variants, we used the difference in the predicted probability for the positive class between the two models (P(is neurite enriched) – P(is soma enriched)) as the final output.

For prediction of the localization behavior of native transcripts we predicted the probability of neurite localization (as described for the test set above) for all potential 3′UTR tiles of a gene with length 150 bp and a step size between tile starting points of 50 bp (i.e. tile 1 corresponds to 3′UTR positions 1–150, tile 2 to 3′UTR positions 51–200, etc.). To capture the positive contributions to neurite localization, we calculated from all individual tiles of a 3′UTR the median prediction probability of the model predicting neurite localization. From this we subtracted the maximal prediction probability of the model predicting soma localization, accounting for the fact that according to our results even single soma-restriction signals can overrule other signals promoting neurite localization. We then compared this combined model output to the localization behavior (log FC > 0 or < 0) reported by Taliaferro *et al.* (44).

### General data analysis

For data analysis, we used python 3.7.3 with pandas 0.24.2, numpy 1.16.2, seaborn 0.9.0, scipy 1.2.1, scikit-learn 0.20.3 and shap 0.34.

## RESULTS

### A massively parallel reporter assay for RNA localization in neurons

To dissect the sequence-encoded regulation of RNA localization in a systematic manner we developed a reporter system that would allow us to test tens of thousands of potential regulatory sequences for their ability to drive localization to neurites.

To select candidate sequences to test, we built on earlier studies characterizing the neurite- and soma-enriched transcriptome (13,14,44,45). In general, the overlap in dendritically localizing RNAs reported in these studies is very low (45), which could be due to differences in the experimental system or cell type used. We selected a core set of dendritically localizing RNAs identified in several independent studies (45) and added genes that were found to be enriched in at least one of the published neurite transcriptome datasets (Methods). In addition, we selected 5 RNAs which have consistently been reported as soma-restricted in previous studies (Rragb, St6gal1, Gpr17, Ogt, Pgap1). This resulted in a set of 315 3′UTRs (Supplementary Table S1) which formed the basis for designing a synthetic oligonucleotide library of altogether 47 347 sequences (Supplementary Table S2).

**Figure 1.**
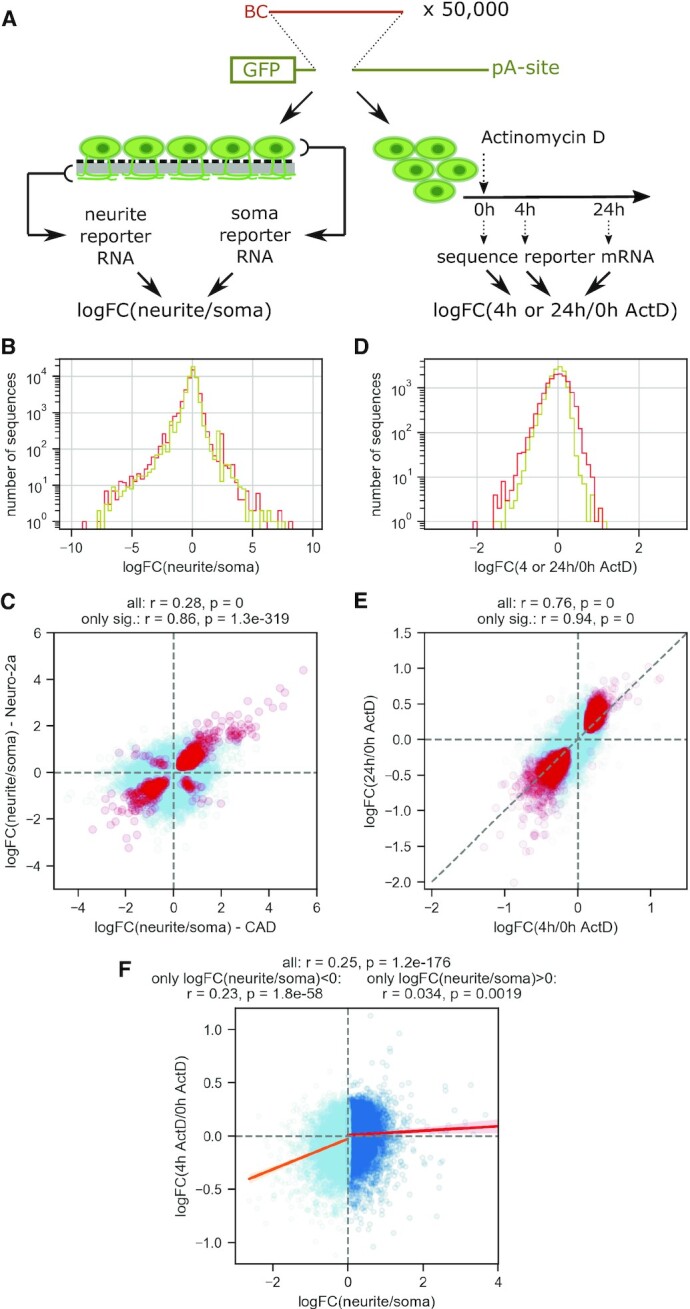
A massively parallel reporter assay for RNA localization in neurons. (**A**) Experimental outline. An oligonucleotide library (containing common primers for amplification (2 × 18 nt), a 5′ barcode (12 nt) and a 150 nt variable 3′UTR region is cloned downstream of the CMV promoter and the *gfp* coding sequence and upstream of a polyadenylation site. For RNA localization measurements (left), the final reporter library is transfected into differentiated neuronal cell lines grown on microporous membranes, and reporter RNA from soma and neurite compartments are collected and sequenced, resulting in a measure for the enrichment of each library variant in the neurite vs. soma compartment. For RNA stability measurements, the reporter library is transfected into differentiated neuronal cell lines, followed by transcription inhibition using Actinomycin D and collection and sequencing of reporter RNA 0, 4 or 24 h later BC: barcode; pA-site: polyadenylation site. (**B**) Density plot of log FC(neurite/soma) measured in CAD (red) and Neuro-2a cells (yellow); *n* = 47 347 for both. (**C**) log FC(neurite/soma) measured in CAD cells are plotted against the logFC measured for the same sequence in Neuro-2a cells, for all sequences tested (light blue, *n* = 47 347) or only those with statistically significant enrichment (neurite or soma, *P* = 0.05) in both cell lines (red, *n* = 3412). (**D**) Density plot of logFC(4 h/0 h Actinomycin D) (yellow) or logFC(24 h/0 h Actinomycin D) (red) measured in CAD cells; *n* = 13753 for both. (**E**) logFC(4 h/0 h Actinomycin D) are plotted against logFC(24 h/0 h Actinomycin D) measured for the same sequences in CAD cells, for all sequences tested (light blue, *n* = 13 753) or only those with statistically significant effect on RNA levels (in 4 h and 24 h, *P* = 0.05; red, *n* = 2542). (**F**) logFC(4 h/0 h Actinomycin D) measurements are plotted against logFC (neurite/soma) as determined in CAD cells; library variants with logFC (neurite/soma) <0 are shown in light blue and the corresponding trend line in orange, library variants with logFC(neurite/soma) >0 are shown in dark blue and the corresponding trend line in red (*n* = 13 753).

Oligonucleotides in our library comprised common primers for amplification, a unique barcode and a 150 nt long variable region containing a 3′UTR sequence (Figure 1A). They were synthesized (Twist Bioscience), PCR amplified, and cloned downstream of a reporter gene (GFP). We transfected the final library into two mouse neuronal cell lines (CAD and Neuro-2a cells), which were differentiated into a neuron-like state by serum starvation (see Methods, Supplementary Figure S1A, (44)). In contrast to primary neurons, these cell lines can be transfected with high efficiency, allowing for high-throughput screening of large synthetic reporter libraries. To allow physical separation of soma and neurites, cells were grown on microporous membranes coated with matrigel on the bottom for 24 h prior to transfection, such that neurites would extend into the bottom compartment while cell bodies remained on top of the membrane. This experimental system has been previously used for sequencing the native soma and neurite transcriptome in CAD and Neuro-2a cells (44) as well as in mESCs differentiated into neurons (13). Using qRT-PCR for known neurite-enriched and soma-restricted genes (Kif1a and Ogt, respectively), we confirmed that our experimental pipeline indeed leads to the expected segregation of neurite and soma transcriptome (Supplementary Figure S1B).

Total RNA from soma and neurite fractions was extracted from three biological replicates one day after transfection and cDNA was synthesized, including the incorporation of unique molecular identifiers (UMI). Reporter library sequences were amplified by PCR and subjected to Illumina sequencing, resulting in UMI counts in all samples for 42 062/47 347 library variants (Supplementary Figure S2). Based on these data, we used edgeR (51) to calculate the relative enrichment (log_2_ fold-change (logFC)) of each library sequence in the neurite and soma fraction, respectively (Methods). The raw enrichment scores are available in Supplementary Table S2.

In both cell lines, most sequences did not show a preferential enrichment in the soma or neurite fraction (Figure 1B). Without active retention in the soma, RNAs can diffuse into (proximal) dendrites; therefore we expect to find most RNAs in both fractions. Significant enrichment of a sequence in the neurite fraction, however, is indicative of active transport, local anchoring in neurites, or local or global protection from degradation. In line with the stochastic nature of the distribution of RNAs without active transport, the general correlation between the two cell lines was modest (Pearson *r* = 0.28, *P* = 0, Figure 1C, light blue). However, when comparing only sequences with significant enrichment in either neurite or soma, the concordance between the two cell lines was much higher (Pearson *r* = 0.86, *P* = 1.3 × 10^−319^, Figure 1C, red), suggesting that the same sequences are actively sorted into neurites (positive log_2_ fold-change) or retained in the soma (negative log fold-change).

We added a unique 12 nt barcode to each library sequence to allow for its identification in RNA-seq reads. We included library variants containing the same sequence to be tested, but different barcodes (see Materials and Methods). Comparing logFC measured in our assay for library variants sharing the same sequence and differing only in the barcode showed good concordance in the direction of the enrichment and suggests that our assay can reliably identify neurite or soma enriched sequences (Supplementary Figure S3AB). To provide additional evidence that the barcode sequence itself does not significantly affect the localization behavior of the full sequence, we repeated our MPRA with a library of reporter constructs containing only the 12 nt barcode, but not the downstream library sequence. Comparing the localization behavior of the barcode-only sequence to the corresponding full library sequence showed no correlation, indicating that the barcode in general does not significantly affect our measurements (Supplementary Figure S3C).

Overrepresentation of specific mRNAs in neurites can be triggered by active transport of these mRNAs or by selective stabilization, increasing their chance of reaching the neurite compartment by passive diffusion. Both of these can be mechanisms actively employed by the cell to achieve neurite localization of specific mRNAs (57). To estimate the effect of passive diffusion on the localization patterns measured in our assay, we assessed the stability of 13 753 library sequences corresponding to native 3′UTR regions by determining their relative abundance after transcription inhibition. Specifically, the 3′UTR sequence library was transfected into differentiated CAD cells (Materials and Methods). Transcription was inhibited by addition of Actinomycin D 24 h later and duplicate samples were collected after 0, 4 and 24 h (Figure 1A, right). Reporter RNA was sequenced as described above and log_2_ fold changes between the relative abundance of each library variant 4 and 24 h compared to 0 h were calculated using edgeR. The resulting data are available in Supplementary Table S2. The distribution of log fold-changes was skewed towards negative values, indicating that destabilizing effects dominated (skew = –0.81, *P* = 2.10 × 10^−253^; Figure 1D). logFC for 4 and 24 h of Actinomycin D treatment were highly correlated (Pearson *r* = 0.76, *P* = 0; for variants with significant effect alone: *r* = 0.94, *P* = 0; Figure 1E), with 24 h treatment leading only to moderately increased fold-changes on average (deviation from the diagonal line in Figure 1E). Measuring relative decay rates for groups of library variants with identical sequence, but different barcodes corroborated the reproducibility of our measurements (Supplementary Figure S4).

We compared the localization behavior to the stability measurement for all the tiles and observed a weak correlation (Pearson's *r* = 0.25, *P* = 1.2 × 10^−176^, Figure 1F). Interestingly, when comparing library sequences with a bias towards soma localization (negative logFC(neurite/soma)) and those with a bias towards neurite localization (positive logFC(neurite/soma)) separately, the correlation was much stronger for soma-biased sequences than neurite-biased sequences (*r* = 0.23, *P* = 1.8 × 10^−58^ versus *r* = 0.034, *P* = 0.0019) This suggests that low stability can prevent an mRNA from accumulating in neurites. On the other hand, increased stability can lead to higher logFC(neurite/soma), but this alone is less predictive of neurite enrichment. We hypothesize that the component of neurite localization that cannot be explained by increased stability is due to other mechanisms such as active transport or local anchoring.

### A subset of neurite enriched transcripts contains focused localization regions in their 3′UTR

**Figure 2.**
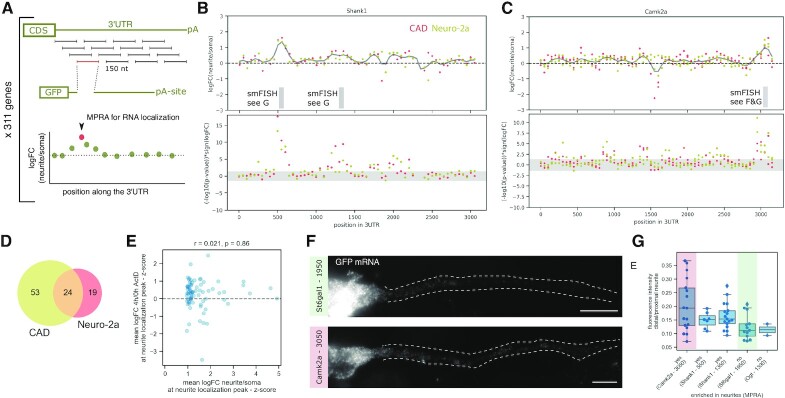
A subset of neurite enriched transcripts contains focused localization regions in their 3′UTR. (**A**) Tiling of the 3′UTR of endogenous genes; starting immediately after the stop codon, segments of 150 nt are tested in isolation, with a distance of 50 nt between two adjacent tiles thereby creating a sequence overlap of 100 nt between adjacent tiles. (**B**, **C**) Measurements (upper panel: logFC(neurite/soma), lower panel: –log_10_(*P*-value) of the effect, multiplied by the sign of the logFC) for tiles along the 3′UTR of Shank1 (B) and Camk2a (C), as measured in CAD (red) and Neuro-2a (yellow) cells; the gray horizontal band denotes the area with *P* > 0.05; the positions for which smFISH validations are shown in panels F and G are indicated below. (**D**) Venn diagram showing the number of unique and overlapping peaks of neurite localization signal identified in the tested 3′UTRs. (**E**) The mean *z*-score of the logFC(4 h/0 h Actinomycin D) at the neurite localization peaks is plotted against the mean *z*-score of the logFC(neurite/soma) as measured in CAD cells; *n* = 77). (**F**, **G**) smFISH images (F) and quantification (G) for selected neurite localizing (Camk2a, Shank1) or soma restricted (St6gal1, Ogt) library sequences, the imaged and quantified individual 3′UTR reporters correspond to the tiles indicated in panels B and C.

In order to map sequences driving localization, we scanned the 3′UTR of 311 genes selected based on our analysis of published datasets of neurite and soma transcriptomes (13,14,44,45) (see above and Materials and Methods). For each gene, our oligonucleotide library contained tiles of length 150 nucleotides (nt), covering the entire 3′UTR with a step size (i.e. distance between start positions of adjacent tiles) of 50 nt. In our MPRA, we tested the resulting 13 753 3′UTR tiles both in CAD and Neuro-2a cells, providing a landscape of localization potential along the 3′UTR (Figure 2A). Based on the localization behavior of these 150 nt tiles, we identified segments of the 3′UTR that showed strong enrichment in the neurite fraction and could constitute the neurite-targeting element mediating localization of the entire native 3′UTR, e.g. in Shank1, Camk2a, Vapb and others (Figure 2B, C, Supplementary Figure S5). We detect such peaks in neurite localization signal in an unbiased way, using a peak detection algorithm to scan the 3′UTRs for peaks with a fold change larger than 1.5 and a mean *P*-value <0.05 for the region of the peak (see Materials and Methods for details). This approach identified 24 peaks shared between CAD and Neuro-2a cells as well as 53 detected only in CAD and 19 only in Neuro-2a cells (Figure 2D). A complete list of these peaks of neurite localization potential is available in Supplementary Tables S3 and S4.

To determine whether the peaks detected in CAD cells can be explained by increased RNA stability, we analyzed the effect of these regions in our stability measurements (Supplementary Table S3). We computed z-scores for the readouts for localization and stability in order to make them more comparable and to estimate to what extent the increased stability conferred by a specific 3′UTR sequence can explain the effect on subcellular localization (Figure 2E). While for some localization peaks with weaker neurite enrichment score increased stability could account for most of the effect, in general there was no correlation between the two readouts and in particular for stronger neurite enrichment regions could not be explained solely by an effect on RNA stability. We therefore postulate that strong localization regions rely on additional mechanisms to achieve neurite enrichment such as active transport.

Among the localization regions detected here are regions that have been previously implicated in RNA localization to neurites. Prior work has analyzed the dendrite-localizing potential in the Camk2a gene and annotated most of it to a sequence stretch at the end of its 3′UTR that is only present in the longest Camk2a isoform (58). Another study found cytoplasmic polyadenylation elements and the RBP binding them (CPEB) to be involved in localization of Camk2a mRNA in rat hippocampal neurons (59). Our Camk2a tile measurements (Figure 2C) are consistent with these prior findings and identify a 200 nt stretch at the very end of the 3′UTR, which is specific to the long Camk2a isoform (58) and contains binding sites for CPEB proteins (Supplementary Table S5).

To validate the results of our MPRA we re-constructed specific reporters carrying either soma restricted or neurite localizing 3′UTR tiles. We transfected these reporters individually into differentiated CAD cells and performed single molecule fluorescence *in situ* hybridization (smFISH) using probes targeting the *gfp* coding sequence (Methods). The localization behavior observed for individual library sequences generally matched the results from the MPRA (e.g. for a localizing Camk2a tile vs. a soma-restricted St6gal1 tile). This difference became particularly evident when comparing distal portions of neurites which are less likely to be affected by passive diffusion of the RNA due to high expression levels (Figure 2F, G).

### Neurite localization can be broadly encoded along the length of the 3′UTR

**Figure 3.**
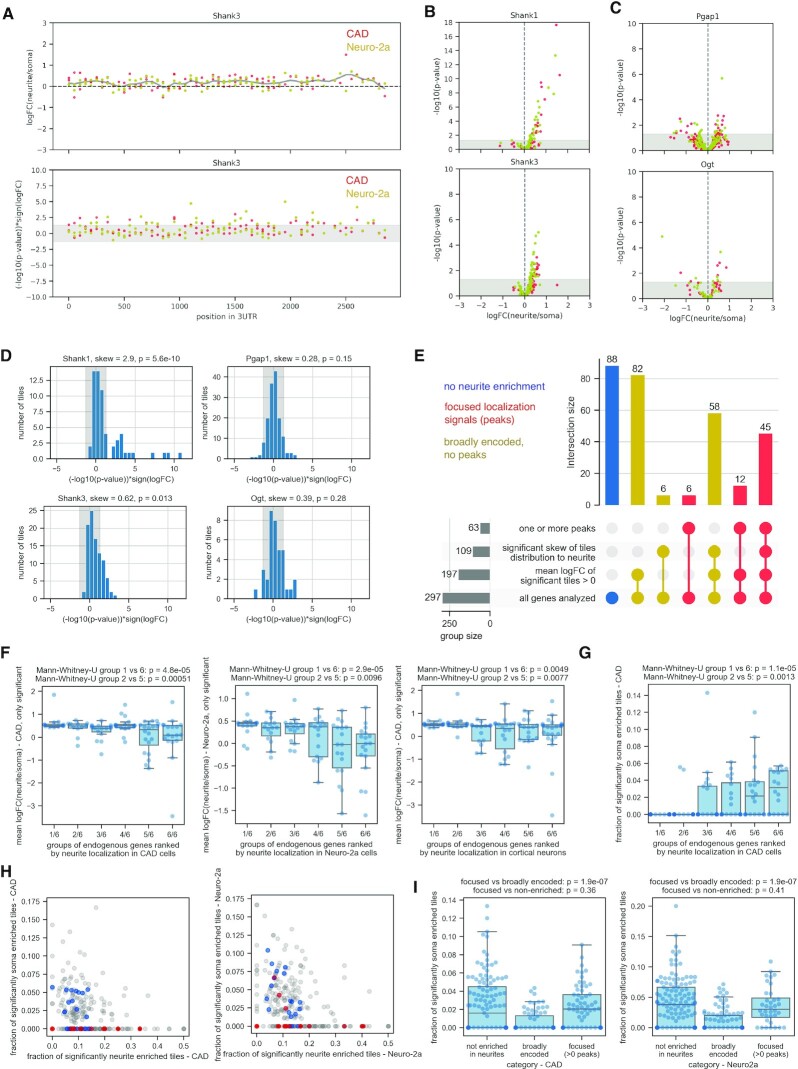
3′UTR fragments recapitulate the localization behavior of the endogenous transcript. (**A**) Measurements (upper panel: logFC(neurite/soma), lower panel: –log_10_(*P*-value) of the effect, multiplied by the sign of the logFC) for tiles along the 3′UTR of Shank3, as measured in CAD (red) and Neuro-2a (yellow) cells. (**B**, **C**) logFC(neurite/soma) measured in CAD (red) and Neuro-2a (yellow) cells are plotted against the –log_10_(*P*-value) of the enrichment, for a gene with a defined region of increased neurite localization potential (Shank1, B, top), a gene with broadly encoded localization potential (Shank3, B, bottom) or genes with no evidence for neurite localization in endogenous RNA-seq data (C). (**D**) The distribution of the *P*-values of the enrichment scores of all tiles of the indicated gene (with the sign indicating the direction of the logFC) is shown; the statistics (skew and associated p-value) given above are used in panel E for classification of genes. (**E**) Upset plot showing the number of genes falling in the indicated categories and the size of the overlap between them (for measurements of logFC(neurite/soma) in CAD cells). (**F**) Each data point represents the mean logFC(neurite/soma) of all tiles corresponding to a segment taken from the same native 3′UTR and showing statistically significant enrichment in any compartment (neurite or soma, *P* = 0.05); the genes are ranked in groups of 20 based on their logFC(neurite/soma) from highest (‘1/6’) to lowest presence in neurites (‘6/6’) as measured by Taliaferro *et al.* (44) for CAD (left), Neuro-2a cells (middle) or cortical neurons (right). (**G**) Each data point represents the fraction of all tiles corresponding to a segment taken from the same native 3′UTR that showed statistically significant enrichment in the soma compartment in CAD cells; the genes are ranked in groups of 20 based on their logFC(neurite/soma) from highest (‘1/6’) to lowest presence in neurites (‘6/6’) as measured by Taliaferro *et al.* (44) for CAD cells. (**H**) The fraction of tiles with significant enrichment in neurites out of all tiles measured for a specific gene (x-axis) is plotted against the fraction of tiles with significant enrichment in soma out of all tiles measured for the same gene (y-axis) in CAD (left) and Neuro-2a cells (right); red indicates genes belonging to the group with the highest presence in neurites (‘1/6’ in panels F and G), blue indicates genes belonging to the group with the lowest presence in neurites (‘6/6’ in panels F and G), as measured by Taliaferro *et al.* (44) for CAD (left) or Neuro-2a cells (right). I. The fraction of tiles with significant enrichment in soma out of all tiles measured for a specific gene is plotted separately for genes according to the categories in panel E (not enriched in neurites, broadly encoded neurite localization potential or focused localization signals (>0 peaks)), for CAD (left) and Neuro-2a cells (right).

While the 3′UTRs described above indicated that the localization potential is encoded by a defined region, in other cases the localization potential was less clearly localized; instead, many 3′UTR regions showed a moderate tendency for neurite enrichment, such as in the case of Shank3, Cntn2 and others (Figure 3A, Supplementary Figure S6). We hypothesize that the localization potential in these, or potentially in most, RNAs is broadly encoded, with many small contributions making up the net localization behavior of the native 3′UTR.

To corroborate this hypothesis we analyzed the localization behavior of the neurite-enriched tiles mapping to a specific gene, without taking into account whether they map to one defined localization region or whether they are spread out over the transcript. This showed no visible difference between genes with a distinct localization element (Figure 3B, top; Supplementary Figure S7, left) and genes with broadly encoded localization potential (Figure 3B, bottom; Supplementary Figure S7, middle). Both groups exhibited a similar tendency for significant positive logFCs of their 3′UTR tiles. In contrast, soma-restricted RNAs showed less significant enrichment at the level of individual tiles, and if a gene contained a tile with enrichment in neurites then it also contained one enriched in the soma fraction, leading to no net enrichment (Figure 3C, Supplementary Figure S7, right, Supplementary Figure S8).

To allow us to quantify the cumulative behavior of tiles of the same native 3′UTR, we computed a number of measures: the mean logFC (neurite/soma) for all tiles or only for those with significant enrichment in one compartment, the fraction of tiles with significant soma or neurite enrichment (based on Figure 3BC) and the skew of the distribution of enrichment of individual tiles (*P*-value × sign(logFC), Figure 3D). The results for all tested genes with a 3′UTR at least 350 nt in length and at least 10 analyzed tiles are summarized in Supplementary Table S6 (229 genes out of the 315 genes included in the library).

In summary, we observed that the analyzed genes fall into three groups: Genes of the first group do not show any evidence for neurite localization on the level of tiles. This could be because they indeed do not localize to neurites (at least in the cells and conditions analyzed) or they rely on larger localization regions which our 150 nt tiles cannot adequately reproduce (52 genes in CAD and 55 in Neuro-2a, blue bars in Figure 3E and Supplementary Figure S9). The second group contains genes with at least one dominant peak in our neurite targeting assay, corresponding to a distinct localization element (61 genes in CAD and 35 in Neuro-2a, red bars in Figure 3E and Supplementary Figure S9). Genes of the last group show enrichment in neurons according to one or more of our criteria (mean logFC (neurite/soma) of tiles with significant effect, significant skew in the distribution of all tiles), but do not contain a clearly defined peak of neurite localization potential (116 genes in CAD and 139 in Neuro-2a, dark yellow bars in Figure 3E and Supplementary Figure S9). Genes with a dominant peak in neurite localization potential often also contain other regions exhibiting neurite enrichment (e.g. Shank1, Figure 2B), such that the categorization of a gene as having a focused localization signal does not imply that all the localization information is restricted to this peak. The concept of broadly encoded localization potential therefore can also apply to this type of localizing mRNAs.

### 3′UTR fragments recapitulate the localization behavior of the endogenous transcript

We then compared the localization behavior of the tiles as measured in our assay to the localization of the entire native 3′UTR as measured using a similar experimental setup and the same neuronal cell lines (44) as well as primary cortical neurons. Specifically, we grouped the 128 genes shared between the two studies into six bins based on neurite localization of their endogenous mRNA in CAD or Neuro-2a cells (44) and compared the different measures computed based on our MPRA-based measurements of neurite/soma enrichment of individual tiles between the groups. The mean of the logFC of all tiles mapping to a gene and showing significant enrichment in either the neurite or the soma fraction was indicative of the localization behavior of the corresponding native transcript (Figure 3F). This was not the case when limiting this analysis to the tiles with the most significant effect (Supplementary Figure S10A), suggesting that localization behavior is the sum of many contributions. Interestingly, the presence of a soma-enriched tile was a strong predictor of the localization behavior, as most strongly neurite localizing 3′UTRs did not contain a single tile exhibiting soma enrichment (Figure 3G, Supplementary Figure S10B). We do observe co-occurrence of neurite and soma enriched tiles in the same 3′UTR, with most soma-restricted genes containing tiles that by themselves can promote neurite localization (Figure 3H). Interestingly, genes with a focused localization region (red in Figure 3E) seem to be less sensitive to the presence of a soma-enriched tile than genes with broadly encoded localization potential without a clear peak (orange in Figure 3E), probably because a strong localization element is able to overcome the negative effect of a soma-enriched sequence element (Figure 3I). Taken together, these data indicate that the localization behavior of a transcript is determined by integrating many effects, potentially with small individual sizes.

### Single RBP motifs can only prevent, but not enforce neurite localization

**Figure 4.**
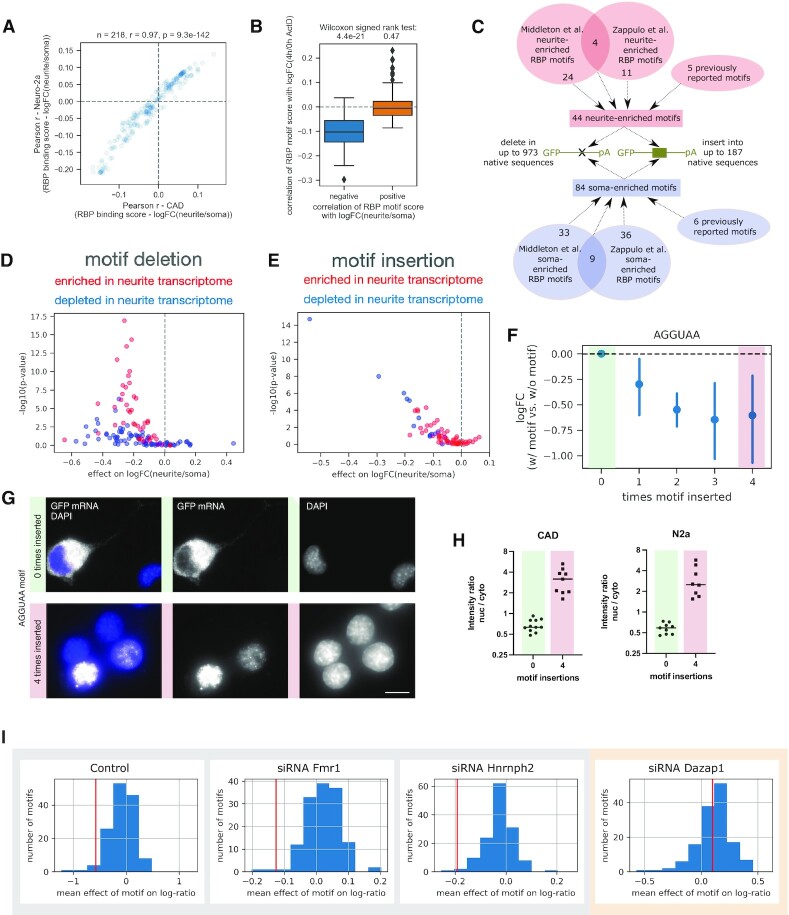
Single RBP motifs can only prevent, but not enforce neurite localization. (**A**) Pearson correlation coefficients between cumulative binding scores of RBPs in all library sequences taken from native 3′UTRs and logFC(neurite/soma) as measured in CAD cells is plotted against Pearson correlation coefficients between the same cumulative RBP scores and logFC(neurite/soma) as measured in Neuro-2a cells. (**B**) The distribution of Pearson correlation coefficients between cumulative binding scores of RBPs and logFC(4 h/0 h Actinomycin D) is shown separately for RBPs showing negative or positive correlation of their cumulative motif scores with logFC(neurite/soma) in CAD cells. (**C**) Schematic of the bioinformatic analysis of available RNA-seq datasets to identify RBP motifs enriched in the soma or neurite transcriptome; these motifs were then either mutated in native 3′UTRs or inserted into native 3′UTR contexts. (**D**) Each data point shows the mean effect on logFC(neurite/soma) in CAD cells for deletion of a specific motif in up to 973 native sequences, plotted against the associated p-value (Wilcoxon signed-rank test), for motifs identified as being enriched in neurite (red) or soma (blue) RNA-seq datasets (Middleton *et al.*, Zappulo *et al.*). (**E**) Each data point shows the mean effect on logFC(neurite/soma) for insertion of a specific motif in up to 187 native sequences in CAD cells, plotted against the associated *P*-value (Wilcoxon signed-rank test), for motifs identified as being enriched in neurite (red) or soma (blue) RNA-seq datasets (Middleton et al., Zappulo *et al.*). (**F**) Mean effect of the AGGUAA motif on logFC(neurite/soma) when inserted in a sequence 0–4 times; *n* = 133, 20, 153, 16 and 17. (**G**, **H**) smFISH images (G) and quantification of nucleus/cytoplasmic fluorescence intensity (H) for two individual 3′UTR reporters without (green) or with four AGGUAA motifs inserted (red, see corresponding MPRA data in panel F). (**I**) Distribution of mean effect sizes of all motifs inserted in native 3′UTRs in control and different RNAi conditions; the red vertical line represents the mean effect of the AGGUAA motif in the different conditions.

A number of RBPs have been implicated in regulating the intracellular localization of mRNAs, e.g. by functioning as an adaptor linking a specific transcript to molecular motors. To analyze the effect of RBP motifs in our reporter RNA library on their neurite or soma localization, we computed a cumulative binding score for 184 RBPs for all library sequences (RBP motifs were based on the RNAcompete dataset (52)). Using regression analysis, we estimated the effect of RBP motifs on neurite and soma localization as measured in our assay. The effect of RBP motifs was highly consistent between CAD and Neuro-2a cells (Pearson's *r* = 0.97, *P* = 9.3 × 10^−142^, Figure 4A). 71 RBPs showed significant positive correlation with logFC(neurite/soma), suggesting that they might promote neurite localization (Supplementary Table S7). Among the most significant hits were also RBPs that have already been implicated in RNA localization, such as Muscleblind (Mbnl) and members of the ELAV family. 101 RBPs showed significant negative correlation with logFC(neurite/soma), suggesting that they might prevent neurite localization or promote soma retention (Supplementary Table S8). We repeated the regression analysis for our measurements of relative RNA decay rates for the same set of library sequences (Supplementary Table S9) and compared the results with those obtained with the RNA localization readout (Figure 4B). As a group, RBP motifs that showed negative correlation with neurite localization were, in many cases, also negatively correlated with RNA stability (*P* = 4.4 × 10^−21^, Wilcoxon's signed rank test), suggesting that a highly overlapping set of sequence elements promotes both soma retention and RNA decay. On the other hand, RBP motifs showing positive correlation with neurite localization did not show a clear trend of being associated with higher or lower stability of the mRNA (*P* = 0.47, Wilcoxon's signed rank test). In particular, A/U-rich elements are very prominent among the motifs negatively correlated with both neurite localization and RNA stability, in line with their known destabilizing function (60). Performing an enrichment analysis for RBP motifs on neurite and soma localizing tiles confirmed the results from our regression-based analysis and yielded a similar set of enriched RBP motifs (Supplementary Tables S10 and S11). The overlap in motifs associated with soma restriction and reduced mRNA stability is consistent with the comparison of our localization and stability measurements (Figure 1F), pointing at a strong functional connection between reduced stability of an RNA and its soma restriction, but much less so for increased stability of an RNA and its neurite enrichment.

In order to functionally test the influence of RBP binding sites on RNA localization to neurites, we created an additional library of 34 236 sequences, which was cloned and tested in the same experimental pipeline as above. Here, we used 5841 150 nt regions taken from the 3′UTR of 230 genes to test the potential of RBP binding sites to drive localization of the transcript. These 230 genes were a subset of the 315 genes chosen as the basis for the library design (see above and Methods) and selected to include 3′UTRs of both neurite-localizing and soma-restricted transcripts based on previously published data (13,14,44,45).

We analyzed existing neurite and soma transcriptomic data (13,45) to identify RBP motifs preferentially found in neurite- or soma-enriched transcripts (Figure 4C, Supplementary Table S12). To test if these RBP binding sites are required for neurite localization or soma retention, we selected 125 RBP binding sites found in our analysis to be enriched in soma or neurite-localizing transcripts (Figure 4C, Supplementary Table S12) and mutated occurrences of these motifs in 5841 native 3′UTR sequences (Methods, see Supplementary Table S2 for a complete list of all the sequences tested). Up to 25 different RBP binding sites were mutated on any specific tile of endogenous sequence, but every RBP binding site deletion was tested individually, thereby yielding up to 25 sequence variants for each endogenous sequence. We then obtained measurements of neurite/soma enrichment for the wild-type sequence, along with variants of the same sequence in which all instances of one RBP binding motif are mutated. Comparing each mutated sequence to its wild-type sequence yielded a readout of the effect of mutation of a given motif on the localization potential. As we perform this sequence alteration in up to 973 native sequences in parallel, we obtain an average effect of motif deletion that is independent of the specific context. Mutation of neurite-enriched motifs often had a significant negative effect on neurite localization, while mutation of soma-enriched motifs did not lead to increased neurite localization (Figure 4D, Supplementary Figure S11A). In some cases mutation of soma-enriched motifs even had a negative effect on logFC(neurite/soma) (Figure 4D), probably due to the fact that any introduced sequence change can affect also the accessibility of other (potentially neurite localization-promoting) sequence elements close by for example through changes in the secondary structure. Comparing the effect of motif mutations between CAD and Neuro-2a cells showed correlation between the cell lines for the mutation of neurite-enriched motifs (Pearson *r* = 0.37, *P* = 0.013, Supplementary Figure S11B, upper panels), providing further evidence that these motifs are required for RNA localization in both cell lines. We validated the effect of motif deletion by performing smFISH on a pair of otherwise identical sequences, one wild-type and one mutant (UCUUCU replaced by random sequences). In our MPRA, the wild-type sequence exhibits significant enrichment in the neurite fraction (log_2_FC = 1.22 (CAD, *P* = 0.00034) and 0.97 (Neuro-2a, *P* = 0.0049), respectively), which is abrogated by UCUUCU mutations (log_2_FC = –0.57 (CAD, *P* = 0.12) and 0.38 (Neuro-2a, *P* = 0.10), respectively). Accordingly, smFISH signal in distal versus proximal neurites was significantly lower in the mutant compared to wild-type (Supplementary Figure S11C).

To test if these RBP motifs can actively drive localization by themselves, we inserted 71 neurite- or soma-enriched RBP binding sites into up to 187 native contexts (see Supplementary Table S2 for a complete list of the resulting sequences and their localization and stability readouts). We then measured their effect on the localization behavior of the transcript by comparing library sequences with or without the motif. In general, motif insertions could only shift localization towards soma, not towards neurite (Figure 4E, Supplementary Figure S11D), with clear differences between motifs which we found in our analysis of endogenous RNA-seq data to be enriched in neurite and soma, respectively (Supplementary Figure S11E; CAD: *P* = 2.7 × 10^−5^, Neuro-2a: *P* = 0.0049, Mann–Whitney *U* test). Comparing motif effects between CAD and Neuro-2a cells (Supplementary Figure S11B, lower panels) showed a good correlation between the cell lines for the insertion of soma-enriched motifs (Pearson *r* = 0.93, *P* = 4.9 × 10^−7^), but not for neurite-enriched motifs (Pearson *r* = 0.25, *P* = 0.059), indicating that the effect of the soma-enriched motifs is robust between cell lines.

Some soma-enriched RBP motif insertions had highly significant effects on localization behavior, with AGGUAA showing the strongest negative effect, both in CAD as well as N2a cells (Supplementary Figure S11D,F). This motif has been reported previously to be found preferentially in soma-enriched transcripts (44). The effect increased with the number of binding sites introduced (Figure 4F). Comparing the distribution of neurite/soma enrichment of sequences with or without the AGGUAA motif introduced showed that this motif could not only prevent localization of the RNA to neurites, but often resulted in enrichment in the soma fraction (Supplementary Figure S11F). To elucidate the mechanism by which AGGUAA leads to soma enrichment, we constructed individual reporter constructs of a sequence with or without four copies of the AGGUAA motif introduced. We performed smFISH as described above and observed a striking enrichment of the AGGUAA-containing reporter in the nucleus (Figure 4G, H).

AGGUAA is a potential binding site for the nucleocytoplasmic shuttling RBP Dazap1. To determine whether potential Dazap1 binding sites could more generally affect localization potential, we computed for every 3′UTR sequence tested in our MPRA a cumulative binding score for Dazap1 based on position weight matrices obtained by RNAcompete (collected in ATtRACT (61)). We indeed observed a negative correlation between Dazap1 binding scores and logFC(neurite/soma) across all sequences tested (Supplementary Figure S11G; Pearson *r* = –0.073, *P* = 2 × 10^−51^; Spearman rho = –0.086, *P* = 2.3 × 10^−70^). To further strengthen the link between Dazap1 and soma restriction, we downregulated Dazap1 (along with other RBPs whose binding sites were enriched in soma-restricted or neurite-localized genes, Figure 4C, Supplementary Table S12). While the negative effect of introducing the Dazap1 motif on logFC(neurite/soma) was present in the control and other RNAi conditions, it was lost upon knock-down of Dazap1 (Figure 4I). This indicates that nuclear retention mediated by Dazap1 is one mechanism underlying soma enrichment and exclusion from neurites.

### A synthetic sequence can drive RNA localization to neurites

**Figure 5.**
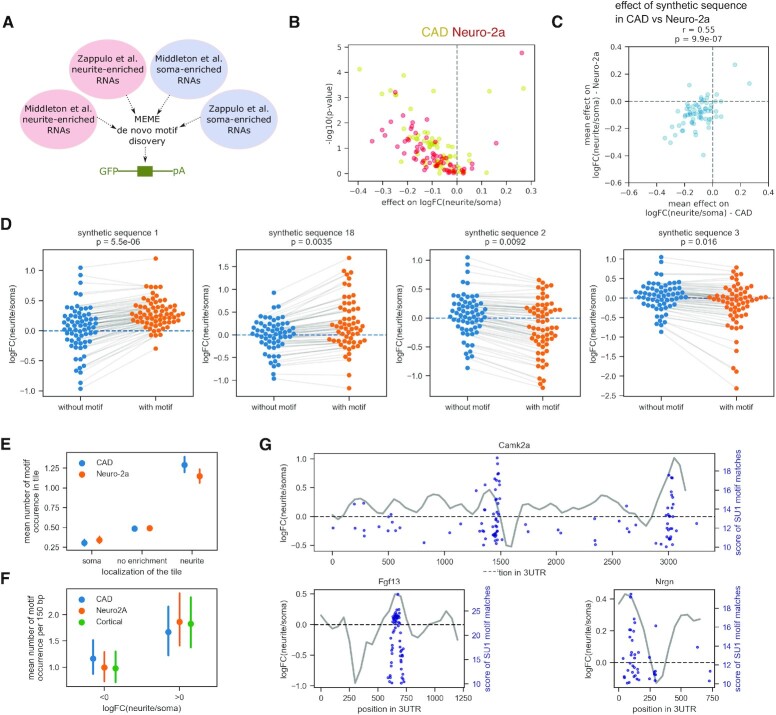
Synthetic sequences can drive RNA localization to neurites. (**A**) Schematic of the bioinformatic analysis of available RNA-seq datasets to identify consensus sequences enriched in the soma or neurite transcriptome; these motifs were then inserted into native 3′UTR contexts. (**B**) Each data point shows the mean effect on logFC(neurite/soma) for insertion of a synthetic sequence in up to 69 native sequences, plotted against the associated p-value (Wilcoxon signed-rank test), in CAD (red) and Neuro-2a (yellow) cells. (**C**) The mean effect on logFC(neurite/soma) for insertion of a synthetic sequence in CAD cells is plotted against the effect of the same motif in Neuro-2a cells. (**D**) Distribution of logFC(neurite/soma) for native 3′UTR sequences without (blue) or with (orange) insertion of the indicated synthetic sequence; the line indicates corresponding pairs of sequences without or with motif insertion. (**E**) Mean number of occurrences of the SU1 motif and 95% confidence interval for tiles showing soma enrichment, no enrichment or neurite enrichment in the MPRA. (**F**) Mean number of occurrences of the SU1 motif and 95% confidence interval for genes showing a bias towards soma (logFC(neurite/soma)<0) or neurite localization (logFC(neurite/soma)>0), as measured by Taliaferro *et al.* (44) for CAD (blue), Neuro-2a cells (orange) or cortical neurons (green). (**G**) The grey line shows the running average of logFC(neurite/soma) of adjacent tiles as measured in CAD and Neuro-2a cells, for all positions along the 3′UTR of the indicated genes; the blue dots indicate matches to the SU1 consensus sequence, the x-axis denotes the start position of the match, the right (blue) y-axis denotes the score of the match.

Our results on insertion or deletion of known RBP motifs suggest that a single motif cannot drive localization to neurites by itself. We therefore aimed to build and test longer synthetic 3′UTR sequences. We generated consensus sequences based on the 3′UTRs of neurite-localized and soma-restricted RNAs identified by Middleton *et al.* and Zappulo *et al.* (Figure 5A). We used MEME Suite to discover ungapped motifs in each of the sets that were enriched over the complementary compartment (i.e. neurite localized transcripts vs. soma localized transcripts; Materials and Methods). The consensus sequences of these motifs were in general very degenerate (Supplementary Table S13). We then chose the best possible matches of the 20 top consensus sequences (for each compartment). We refer to these sequences as synthetic 3′UTR sequences in the sense that they were derived from the analysis of transcriptomic data, but the specific sequences generated here (the best possible matches to the consensus) do not appear anywhere in the mouse genome. We avoided sequences with long homopolymeric stretches as they would pose a problem for synthesis and subsequent steps. Matches for the remaining 66 consensus sequences were then embedded within 69 native contexts and their distribution between neurite and soma fractions was determined together with the rest of the library reporters. Unlike in the case of single RBP motifs whose introduction to a 3′UTR could only promote soma restriction, here we also identified longer synthetic sequences that resulted in increased enrichment in the neurite fraction (Figure 5B). The localization behavior of these sequences was similar in both cell lines used (Figure 5C, Pearson *r* = 0.55, *P* = 9.9 × 10^−7^). One of the synthetic sequences showed particularly robust localization to neurites, irrespective of the sequence context (Figure 5D, synthetic sequence 1). In other cases, the sequence context seemed to affect the localization behavior more; here, only a subset of insertion events led to efficient dendritic localization of the sequence containing the inserted synthetic sequence (Figure 5D, synthetic sequence 18).

We hypothesized that if introduction of synthetic sequence 1 consistently biases any reporter RNA sequence to neurites, endogenous matches of the underlying consensus sequence SU1 (synthetic UTR 1, Supplementary Figure S12A) in native 3′UTRs might contribute to neurite localization of the corresponding RNA. We therefore scanned the 13,753 sequences of our tiling library of native 3′UTR sequences for occurrences of the SU1 consensus sequence (not for exact matches of synthetic sequence 1 used for functional testing above). The mean number of matches per tile was strongly associated with the localization behavior, with neurite-enriched tiles containing on average more matches to the SU1 consensus sequence (Figure 5E). We extended this analysis to full-length native 3′UTRs and compared the number of motif occurrences (per sequence length) between genes showing a bias for soma or neurite localization in CAD, Neuro-2a and cortical neurons, using a dataset that was not used to generate these consensus sequences (44). Also here, neurite localizing RNAs in all three cell types contain on average more matches to the SU1 consensus sequence (Figure 5F). When inspecting the location of the matches to the SU1 consensus on the 3′UTR, we noticed that in 15 out of 77 cases SU1-like sequences colocalized with peaks of neurite localization signal. In the case of Camk2a, Fgf13 and Rnf165 and in one of the peaks in the Shank1 3′UTR, SU1 motifs were highly enriched at the localization peak compared to the rest of the transcript (Figure 5G and Supplementary Figure S12B), suggesting that matches of the SU1 consensus might be a common constituent of native neurite localization regions.

### Dissecting the mechanisms of neurite localization of the SU1 sequence

**Figure 6.**
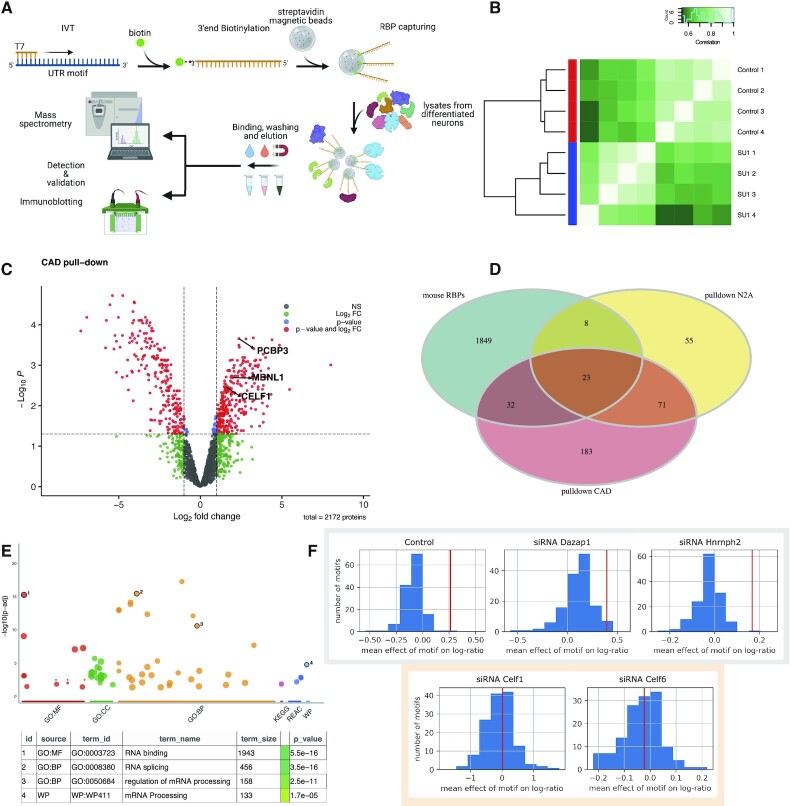
Mass spectrometric analysis reveals proteins that bind to the SU1 synthetic transcript and mediate its neurite localization potential. (**A**) Schematic of the SU1 *in vitro* transcription and pulldown experiments for mass spectrometric analyses. (**B**) Heatmap of across-sample spearman correlations in CAD cells. (**C**) Differential protein pulldown analysis in CAD cells. (**D**) Venn diagram showing the intersection of significantly pulled-down proteins with the SU1 probe in CAD and N2A cells with an annotated list of canonical mouse RBPs (63). (**E**) Manhattan plot of gene set enrichment analysis (g:Profiler,(68)) of the significantly pulled-down proteins (both cell lines); gene sets of particular interest are highlighted in the legend. Dot size refers to the member number of the gene set. The x-axis represents functional terms that are grouped and colour-coded by data sources. The y-axis shows the adjusted enrichment p-values in negative log10 scale. (**F**) Distribution of mean effect sizes (mean change in logFC(neurite/soma) in pairs of sequences with or without a specific motif introduced) of all motifs inserted in native 3′UTRs in control and different RNAi conditions; positive values indicate that a motif on average biases localization to neurites, negative values indicate that a motif on average biases localization to soma, values around 0 indicate that the motif has no effect; the red vertical line represents the mean effect of the SU1 motif in the different conditions; the p-value (Wilcoxon signed-rank test) for the effect of SU1 motif insertion in the respective condition is shown above.

To investigate by which mechanism this synthetic 3′UTR (synthetic UTR sequence 1, SU1; Figure 5D, left; Supplementary Table S14) can localize to neurites, we aimed to identify proteins interacting with this sequence using pull-down and mass spectrometry. As a control, we selected a pool of four sequences of comparable length that were tested in our MPRA and did not show enrichment in neurites (Supplementary Table S12). Both the SU1 and the control sequence pool were PCR-amplified with a T7 promoter sequence, *in vitro* transcribed, biotinylated, and used as baits for pulldowns against total protein lysates from CAD and Neuro-2a cells (Figure 6A). We analyzed the proteomic composition with liquid chromatography and tandem mass spectrometry (LC-MS/MS, methods) and detected a total number of 2172 proteins in CAD cell eluates and 2038 proteins in Neuro-2a cells, respectively. The resulting proteomic measurements of four SU1 eluates clustered separately from four control eluates in a heatmap of spearman correlations between all samples (Figure 6B). This indicates that the variation within replicate groups is considerably smaller than the observed variation of interest across groups. We performed differential protein expression analysis with Maxquant between the SU1 and the control groups, separately for each cell line (Figure 6C, Supplementary Figure S13). In CAD cells, 549 proteins exhibited a differential expression between SU1 and control groups with an adjusted *P*-value <0.05 (Supplementary Table S15). In Neuro-2a cells, 706 proteins were differentially detected between the groups (Supplementary Table S16). We subjected the positively enriched proteins that were detected on the SU1 samples in both cellular contexts to protein list profiling with gProfiler2 (62) and obtained several strongly enriched terms that reflect RNA binding and processing (Figure 6D).

We then set out to identify RNA binding proteins that could mediate the observed neurite localization pattern of SU1 in our proteomic dataset. To this end, we intersected the significant positively enriched proteins from both cell lines with a database of annotated RBPs (63) and retrieved a set of 23 RBP candidates for further investigation (Supplementary Table S17, Figure 6D, E). We complemented the biochemical study of SU1 binders with an *in silico* analysis of RBP motif presence using RBPmap (64) (Supplementary Table S18). Several RBP candidates from our intersection analysis (Figure 6E) exhibited a significant binding motif enrichment in the SU1 sequence: Celf1 (*z*-score 2.643, *P* = 4.11 × 10^−3^), Mbnl1 (*z*-score 1.974, *P* = 2.42 × 10^−2^), and Rbm38 (*z*-score 4.333, *P* = 7.35 × 10^−6^). The combined evidence from biochemical pulldowns and binding motif analyses led us to choose Celf1 for functional validation with RNA interference assays. Additionally, we abrogated Celf6, an RBP that was predicted to bind to the same SU1 sequence stretch as Celf1 by RBPmap. The abrogation of both Celf1 and Celf6 led to a loss of the localization potential of SU1 when compared to non-targeting control, Dazap1, or Hnrnph2 RNAi experiments (Figure 6F), indicating that these proteins are necessary for SU1 subcellular localization.

### MPRA-trained models predict subcellular localization of endogenous 3′UTRs

**Figure 7.**
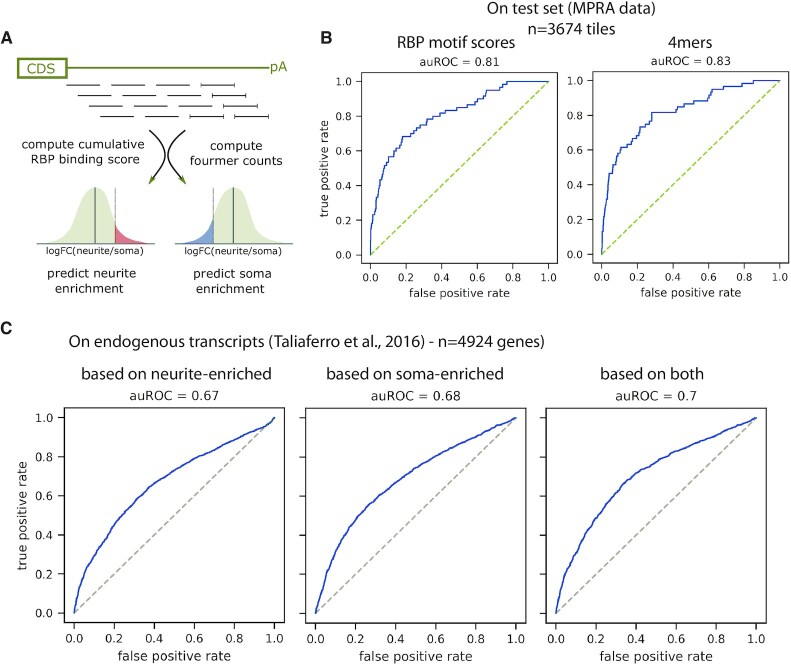
MPRA-trained models predict subcellular localization of endogenous 3′UTRs. (**A**) Outline of the prediction strategy. (**B**) Receiver operating characteristic (ROC) curves showing performance of a classifier trained on 90% of all library sequences passing filtering (at least 500 UMI reads) and testing on the remaining 10% (*n* = 3674; held-out test data), using cumulative RBP motif scores or fourmer counts as features; auROC: area under the ROC curve. (**C**) Receiver operating characteristic (ROC) curves showing performance of a classifier trained on 90% of all library sequences passing filtering and testing on 4924 genes for which neurite/soma distribution in CAD cells has been determined previously (44); the prediction constitutes the combined prediction for 150mers from the native 3′UTR, based on models predicting neurite enrichment (left), soma enrichment (middle) or the combined output of both (right).

Our large dataset of neurite/soma distributions of 3′UTR reporter sequences provides a starting point for deciphering the regulatory logic of RNA localization and for prediction of the localization behavior of novel sequences. Since our data suggested that both neurite as well as soma enrichment can be actively mediated by sequence motifs, we trained two classifiers on 90% of our data (XGBoost, gradient boosting decision trees, see Materials and Methods): One to discriminate between sequences driving significant neurite enrichment (*P* < 0.05) and all others and one to discriminate between sequences driving significant soma enrichment (*P* < 0.05) and all others (Figure 7A). The remaining 10% served as the test set and were not used at any point in building the model.

RBPs are thought to be the *trans-*acting factors mediating localization to neurites. We therefore computed cumulative binding scores for a set of 218 RBPs for which binding sites have been identified (RNAcompete (52)) and used these as features to train our models. As an alternative, unbiased approach, we used counts of all possible fourmers in the sequence as features. We scored performance of prediction algorithms and parameter settings on the training set by cross-validation and then trained both predictors on the entire training set (33 057 sequences). The combined output of both models was able to predict the localization behavior of unseen variants with high accuracy (Figure 7B; area under the receiver operating characteristics curve (auROC) was 0.81 for motif scores and 0.83 for fourmers). Fourmers showed a slightly better performance, indicating that restricting the features to known RBP binding sites might not capture all the relevant information. We used Shapely (SHAP) values (65) for determining the contribution of each feature to the prediction result of every sample (Supplementary Figure S14). This identified sequence elements and RBP binding motifs driving the prediction, providing starting points for follow-up studies validating these findings and investigating a potential role of the corresponding trans-acting factors in mediating neurite or soma enrichment.

According to our data, the localization potential of a native 3′UTR tends to be broadly encoded in the sequence and is not necessarily restricted to one clearly defined localization motif. In order to apply our model to predict the localization behavior of native 3′UTRs we therefore chose first to predict the localization potential of individual tiles of native 3′UTRs, defined the same way as in our library (150 nt in length, 50 nt step size between tiles). For each of these tiles, our models predict the likelihood that it will significantly affect neurite and soma localization, respectively. In line with our observation that the existence of a soma-enriched segment in a 3′UTR sequence can have a dominant-negative effect and prevent localization of the transcript, prediction of neurite localization based on (lack of) somatic enrichment of any tile performed as well as prediction based on a significant dendritic enrichment as the positive class (auROC = 0.68 versus auROC = 0.67; Figure 7C). Combining both prediction strategies slightly improved prediction further (auROC = 0.7). The lower performance of our predictor on endogenous full-length transcripts compared to new 3′UTR reporter sequences not used to train the model (0.68 versus 0.83) shows that our tile-based approach does not capture all the determinants of localization that are present in endogenous 3′UTRs, in particular those ones that depend on sequence elements larger than 150 nt, endogenous expression levels, secondary structure, RNA modifications or the gene's native genomic environment. Nevertheless, these results indicate that in many cases the localization potential of a native 3′UTR can be inferred by estimating contributions of different segments individually.

## DISCUSSION

Mechanism and functional importance of RNA localization have been central topics in biological research over the last decades. Despite numerous insights into the localization of well characterized transcripts, a general understanding of the link between sequence and function is still missing, curbing our ability to predict the effect of sequence alterations on localization dynamics.

Here, we established a novel experimental approach that enables the mapping of RNA sequence to subcellular localization. The systematic nature of our assay and the large number of sequences tested allowed us to investigate the general principles of RNA localization and bring us one step closer to deciphering the sequence-encoded rules of targeting RNAs to neurites. A complementary study using high-throughput assays to identify localization motifs is submitted back-to-back with this manuscript (66).

Our results agree with the existing literature about known 3′UTR-regions that encode neurite-localization potential; The dendritic localization potential of Camk2a was shown to be encoded mostly in its longest isoform (58), involving CPEB (59), and our strongest neurite-localization potential lies within the gene region that is specific to the long isoform and contains binding sites for CPEB proteins.

**Figure 8.**
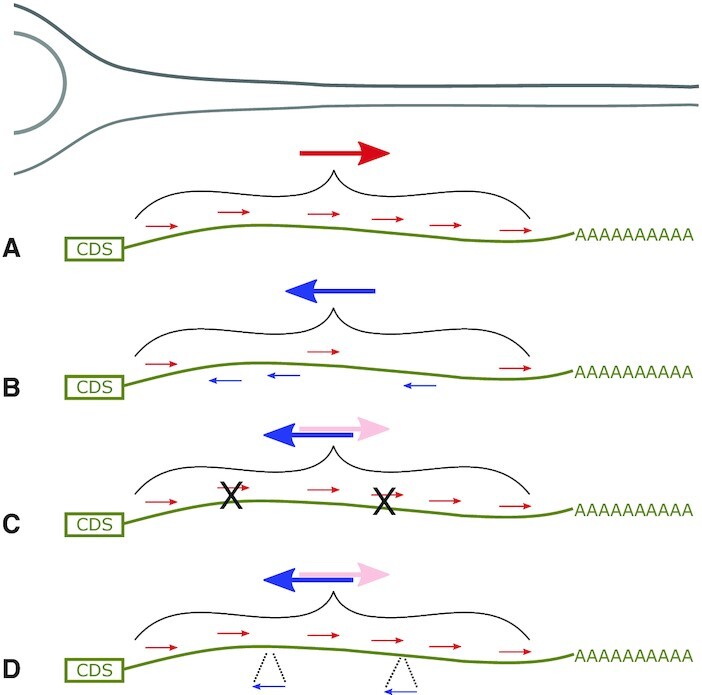
Localization potential as a sum of small contributions. (**A**) Sequence elements promoting neurite localization (small red arrows) encoded along the length of the 3′UTR promote enrichment of a transcript to neurites (large red arrow). (**B**) Contributions from sequence elements promoting neurite (small red arrows) and soma (small blue arrows) neutralize each other, leading to restriction of the transcript to the soma (large blue arrow). (**C**) Mutation (‘X’) of sequence elements promoting neurite localization (small red arrows) can abolish enrichment of the transcript in neurites. (**D**) Introduction of a sequence element promoting soma restriction (small blue arrow) can prevent localization to neurites.

From our measurements of the localization behavior of 13 753 native sequences and 34 236 designed sequence variants the following model emerges: We propose that in most cases the localization potential is broadly encoded along the length of the 3′UTR sequence. While in some genes a defined region with strong potential for neurite localization can be identified, this is not the case for many other transcripts found in neurites. While we cannot rule out that for some of these genes our MPRA is not an adequate tool to identify the localization regions, our results show that the collective localization behavior of all the 3′UTR tiles combined does recapitulate the localization reported for the endogenous transcript. We therefore suggest that many contributions with individual small effect size, for example, RBP binding events, can slightly bias localization of a transcript towards neurite or soma (Figure 8A, B). Combined, these small contributions can drive neurite localization of the entire transcript. This way of encoding neurite localization might be more robust to small sequence changes and more effective in preventing ectopic localization of soma-restricted transcripts. This model is in line with the fact that despite large experimental efforts over the last decades only a small number of focused localization elements could be identified.

We identify 3′UTR sequence regions sufficient to drive localization to neurites; however, as we typically detect more than one 3′UTR region showing neurite localization potential, it remains unclear to what extent each individual region is required for localization of the endogenous transcript. The complexity of the possible regulatory connections does not allow us to determine to what degree cooperativity between neurite-localization promoting elements plays a role, but our data point at antagonism between elements promoting neurite localization and soma retention. Based on our observation that low RNA stability is associated with exclusion from neurites, we hypothesize that one mechanism by which this antagonism manifests itself is by affecting the stability of the mRNA.

Based on these principles, we developed a computational model to predict the localization behavior of a native 3′UTR based on the contribution of small parts. The model trained on our MPRA data was indeed able to discriminate between neurite-localizing and non-localizing endogenous transcripts with good accuracy, corroborating our view of the localization behavior as a sum of different contributions from 3′UTR tiles promoting neurite or soma localization.

At the level of individual 3′UTR tiles, localization to neurites can be abrogated by mutating individual RBP binding motifs (Figure 8C), but it cannot be created by introducing potential RBP binding sites. The preferences for functional binding of an RBP are probably more complex and go beyond the narrow sequence motif. Therefore, merely introducing an RBP binding motif is not sufficient to actively drive translocation of a transcript to neurites.

In contrast, enrichment of an mRNA in neurites can be actively prevented by introducing sequence motifs (Figure 8D). Our results reveal one possible mechanism by which this observation can be explained mechanistically, namely nuclear retention. We identify a potential Dazap1 binding motif as a promoter of nuclear retention and consequently as a strong inhibitor of neurite enrichment. This Dazap1-mediated nuclear retention presented the most drastic effect in our assay, and we postulate that other associations with soma-restricted RBPs underlie our observation of widespread dominant negative activity of soma-enriched motifs on neurite localization. Our parallel assessment of localization potential and RNA stability showed that decreased stability is strongly associated with soma retention, offering an additional mechanism by which the cell can prevent mis-localization of RNAs to neurites. While increased stability also contributes to - or is a prerequisite of - neurite localization, it is not enough to explain the observed localization patterns and is likely complemented by other mechanisms of creating asymmetric intracellular RNA distribution such as active transport or local anchoring.

Since the context-dependent addition of short RBP motifs did not suffice in encoding neurite-localization potential, we attempted to assemble larger de-novo motifs based on observed consensus sequences across endogenous 3′UTRs enriched in neurite and soma transcriptomes. This approach indeed yielded sequences that robustly biased localization to neurites. We then dissected the localization potential of one of our synthetic 3′UTR sequences. Converging evidence from bioinformatic analysis, mass spectrometry and RNAi experiments pointed at a number of prominent RBPs which have not been directly implicated in RNA localization up to date, in particular Celf1 and Celf6. These results show that high-throughput functional testing can reveal novel players of RNA localization and advance our understanding of the complex interplay between RBPs and the transcriptome.

Our experimental strategy allows us to perform high-throughput testing of the localization potential of a sequence, but entails also a number of trade-offs: In most cases, more accurate measurements for any single sequence can be made if that specific sequence was tested in isolation. However, the power of this (or any) MPRA lies in the number of sequences tested. Especially for quantitative comparisons we draw our conclusions based on many sequences being tested, e.g. the same RBP motif being inserted and tested in dozens of sequence contexts. A particularly important point is the choice of model system: As efficient transfection of the large number of reporter constructs is key to obtaining high-quality quantitative data, we chose two neuronal cell lines, CAD and Neuro-2a cells, as model systems. In vivo, localization of certain RNAs can depend on cell identity, neural activity or the tissue context. While these specific features of RNA localization cannot be fully reproduced in a neuronal cell line, we believe that the general principles of RNA localization to neurites will be similar, and predictions from our MPRA can subsequently be tested in a targeted way in primary neurons or other suitable models that are incompatible with the large scale of our MPRA library. We also limit ourselves to the primary sequence of the 3′UTR and do not take into account additional factors such as secondary structure and RNA modification, which could influence RNA localization for example through affecting RNA-RBP interactions or more generally the accessibility of regulatory elements. In addition, the synthesis of a large number of rationally designed sequences limits the length of the sequence that can be tested (to 150 nt in our case). While some more complex localization motifs might be missed by our current MPRA setup, our data show that we can detect many known and novel localization motifs across transcripts. Furthermore, we show that taken together, the 3′UTR tiles tested in our assay recapitulate the endogenous localization behavior of the full transcript.

Many neurological diseases have been linked to dysregulation of RNA localization (67). In the future, the high-throughput approach developed here can enable us to predict and experimentally test how genetic variation affects the localization potential of a sequence. This can reveal the functional consequence of disease-associated genetic variants and thereby highlight new therapeutic strategies. Beyond the model system employed here, this approach can be extended to the study of subcellular RNA localization in other tissues like intestinal epithelia, which will allow for a systematic comparison of regulatory mechanisms of RNA localization.

## DATA AVAILABILITY

Illumina sequencing data generated in this study are available in the NCBI gene expression omnibus (GEO) under accession GSE173098. The mass spectrometry proteomics data have been deposited to the ProteomeXchange Consortium via the PRIDE partner repository with the dataset identifier PXD025492.

## Supplementary Material

gkac806_Supplemental_Files
