# Supplementary material for: A massively parallel reporter assay reveals focused and broadly encoded RNA localization signals in neurons
Source: Nucleic Acids Res. 2022 Sep 26;50(18):10643–64. doi: 10.1093/nar/gkac806 (PMC9561380; doi:10.1093/nar/gkac806)
Supplement: gkac806_Supplemental_Files [file gkac806_supplemental_files.zip › SupplementaryData_NAR_Final/SupplementaryFiguresAndLegends.pdf]

Supplementary Data for:

A massively parallel reporter assay reveals focused and broadly encoded RNA localization signals in neurons

Martin Mikl[1,2*], Davide Eletto[1], Malak Nijim[2], Minkyoung Lee[1], Atefeh Lafzi[1], Farah Mhamedi[1], Orit David[2], Simona Baghai Sain[1], Kristina Handler[1], Andreas E. Moor[1*]

Affiliations:

1. Department of Biosystems Science and Engineering, ETH Zürich, Basel, Switzerland

2. Department of Human Biology, University of Haifa, Haifa, Israel

* Corresponding authors: Martin Mikl

E-mail: mmikl@univ.haifa.ac.il

Andreas E. Moor

E-mail: andreas.moor@bsse.ethz.ch

Supplementary Figure Legends

Figure S1. Differentiated CAD and Neuro-2a cells as model systems for studying RNA localization.

A. Bright field images of CAD cells grown in differentiation medium (day 6). B. qRT-PCR analysis of known soma-restricted and neurite-localizing mRNAs. CAD cells were grown in differentiation medium on microporous membranes and soma and neurite RNA were collected as described for the MPRA. qRT-PCR detection of a known soma-restricted (Ogt) and neurite-localizing (Kif1a) was performed in triplicates in 4 independent biological replicates. The results shown are normalized to Ogt.

Figure S2. Correlations between samples

Pairwise comparisons of UMI counts for the three soma and neurite replicates are shown in the lower left half. The diagonal shows the distribution of read counts for each sample. X- and y-axes denote UMI read counts in log10. The upper right half shows Pearson correlation coefficients between the individual samples.

Figure S3. Barcode control experiments for the RNA localization MPRA

AB. Examples of logFC(neurite/soma) in CAD (A) and Neuro-2a cells (B) for groups with identical native 3'UTR sequence driving soma or neurite localization, but different barcodes, are shown. The y-axis denotes the gene and position in the 3'UTR from which the library sequence has been taken. C. Comparison of logFC(neurite/soma) of 443 full library sequences (containing a 12 nt barcode in addition to the 3'UTR to test) as measured in our MPRA (x-axis) to logFC(neurite/soma) of the same set of library variants containing only the 12 nt barcode but lacking the 150 nt of native sequence to test. Orange denotes sequences for which the full construct showed significant enrichment either in the soma or in the neurite compartment.

Figure S4. Barcode control experiments for the RNA decay MPRA

AB. Examples of logFC(4h/0h ActD) (A) and logFC(24h/0h ActD) (B) for groups with identical native 3'UTR sequence, but different barcodes, are shown. The y-axis denotes the gene and position in the 3'UTR from which the library sequence has been taken. ActD: Actinomycin D.

Figure S5. 3’UTRs with focused localization signals

Measurements (upper panel: logFC(neurite/soma), lower panel: -log10(p-value) of the effect, multiplied by the sign of the logFC) for tiles along the 3’UTR of genes with a region of increased neurite localization potential, as measured in CAD (red) and Neuro-2a (yellow) cells; gray denotes the area with p>0.05.

Figure S6. 3’UTRs with broadly encoded localization potential

Measurements (upper panel: logFC(neurite/soma), lower panel: -log10(p-value) of the effect, multiplied by the sign of the logFC) for tiles along the 3’UTR of genes with broadly encoded localization potential, as measured in CAD (red) and Neuro-2a (yellow) cells; gray denotes the area with p>0.05.

Figure S7. Comparison of the different groups of neuronal 3’UTRs.

logFC(neurite/soma) measured in CAD (red) and Neuro-2a (yellow) cells are plotted against the p-value of the enrichment, for genes with a defined region of increased neurite localization potential (A), genes with broadly encoded localization potential (B) or genes with no evidence for neurite localization in endogenous RNA-seq data (C).

Figure S8. 3’UTRs of non-localizing genes

Measurements (upper panel: logFC(neurite/soma), lower panel: -log10(p-value) of the effect, multiplied by the sign of the logFC) for tiles along the 3’UTR of genes with no evidence for neurite localization in endogenous RNA-seq data, as measured in CAD (red) and Neuro-2a (yellow) cells; gray denotes the area with p>0.05.

Figure S9. Categorization of genes based on the localization pattern of 3’UTR tiles.

A. Upset plot showing the number of genes falling in the indicated categories and the size of the overlap between them (for measurements of logFC(neurite/soma) in Neuro-2a cells). B. As in A, but for the intersection of CAD and Neuro-2a cells (e.g. only genes with localization peaks in both cell types will be counted as having one or more peaks).

Figure S10. The fraction of soma-restricted tiles recapitulates the localization of the corresponding endogenous mRNAs.

A. Each data point represents the maximal logFC(neurite/soma) of all tiles corresponding to the same native 3'UTR (left) in CAD (top and bottom) and Neuro-2a cells (middle) with statistically significant enrichment in any compartment; the groups correspond to bins of 20 genes grouped by decreasing neurite enrichment (from 1 to 6) as measured by Taliaferro et al. (2016) in Cad (top), Neuro-2a cells (middle) or cortical neurons (bottom). B. Each data point represents the fraction of all tiles corresponding to the same native 3'UTR (left) in CAD (middle) and Neuro-2a cells (top and bottom) with statistically significant enrichment in the soma compartment; the groups correspond to bins of 20 genes grouped by decreasing neurite enrichment (from 1 to 6) as measured by Taliaferro et al. (2016) in Neuro-2a cells (top) or cortical neurons (middle and bottom).

Figure S11. The effect of RBP motif deletion and insertion on neurite and soma localization.

A. Each data point shows the mean effect on logFC(neurite/soma) for deletion of a specific motif in Neuro-2a cells in up to 973 native sequences, plotted against the associated p-value (Wilcoxon signed-rank test), for motifs identified as being enriched in neurite (red) or soma (blue) RNA-seq datasets (Middleton et al., Zappulo et al.). B. The mean effect on logFC(neurite/soma) for mutation (top) or insertion (bottom) of an RBP motif (neurite-enriched, soma-enriched or both) in CAD cells is plotted against the effect of the same motif in Neuro-2a cells. C. Quantification of dendritic localization (as determined by smFISH) for a pair of wild-type/mutant sequences: the wild-type sequence corresponds to positions 850-1000 in the Vapb 3'UTR, the mutant is the same sequence with all instances of the UCUUCU motif replaced by random sequences. D. Each data point shows the mean effect on logFC(neurite/soma) for insertion of a specific motif in up to 187 native sequences in Neuro-2a cells, plotted against the associated p-value (Wilcoxon signed-rank test), for motifs identified as being enriched in neurite (red) or soma (blue) RNA-seq datasets (Middleton et al., Zappulo et al.). E. Box plots showing the distribution of the mean effect of motif insertions on logFC(neurite/soma), for motifs identified as being enriched in neurite or soma RNA-seq datasets (Middleton et al., Zappulo et al.). F. Histogram of native 3'UTR sequences without (blue) or with (orange) insertion of the AGGUAA motif in CAD (top) and Neuro-2a cells (bottom). G. The cumulative binding score for Dazap1 is plotted against the measured logFC(neurite/soma) in CAD cells for all the library sequences; Pearson and Spearman correlation and the associated p-values are given above.

Figure S12. Matches of the SU1 consensus sequence in 3'UTRs of neurite-localizing RNAs.

A. Sequence logo of the consensus sequence of the SU1 motif. B. The grey line shows the running average of logFC(neurite/soma) of adjacent tiles as measured in CAD and Neuro-2a cells, for all positions along the 3'UTR of the indicated genes; the blue dots indicate matches to the SU1 consensus sequence, the x axis denotes the start position of the match, the right (blue) y-axis denotes the score of the match.

Figure S13. Mass spectrometric analysis of proteins associated with the SU1 synthetic sequence in Neuro-2a cells.

A. Heatmap of across-sample spearman correlations in Neuro-2a cells. B. Differential protein pulldown analysis in Neuro-2a cells.

Figure S14. Features driving prediction of neurite or soma enrichment.

Effects (as determined using SHAP) of 4mers (top) and cumulative RBP binding scores (bottom) on the model prediction (ranked by their importance for the prediction) for a classifier built on the indicated feature sets and predicting significant neurite (left) or soma (right) enrichment ($p<0.05$). The color denotes the feature value and the position along the x-axis denotes the impact on model output, for each item in the training set.

Figure S15. Confirmation of gene knock-down by RNAi

Mock or siRNA treatment of CAD cells, followed by qRT-PCR analysis of target genes (normalized to a reference gene (Tbp) and the respective mean in the mock condition (DeltaDelta-Ct); data points represent individually transfected and processed wells.

Supplementary Table Legends

Table S1. List of genes used in the library design.

Gene and transcript ID, gene name and genomic coordinates for the 315 genes selected for in depth analysis of their localization potential.

Table S2. List of library variants and MPRA readouts.

All sequences included in the library, including gene name, position in the 3'UTR (relative to the stop codon), introduced sequence changes and MPRA readouts (logFC(neurite/soma) in CAD and Neuro-2a and associated p-values, logFC(4h or 24h/0h Actinomycin D) and associated p-values).

Table S3. List of focused neurite localization regions in CAD

Peak regions of neurite localization signal as determined using an unbiased peak detection algorithm in MPRA data on CAD cells. Gene name, position in the 3'UTR (relative to the stop codon), peak height (logFC(neurite/soma) at the position of the peak), the harmonic mean of the p-value of the neurite enrichment for tiles in the peak region (peak position and immediately adjacent tiles with an overlap of 100 bp), logFC(4h/0h Actinomycin D) and associated p-value for the tiles in the peak region.

Table S4. List of focused neurite localization regions in Neuro-2a

Peak regions of neurite localization signal as determined using an unbiased peak detection algorithm in MPRA data on Neuro-2a cells. Gene name, position in the 3'UTR (relative to the stop codon), peak height (logFC(neurite/soma) at the position of the peak) and the harmonic mean of the p-value of the neurite enrichment for tiles in the peak region (peak position and immediately adjacent tiles with an overlap of 100 bp).

Table S5. RBP motifs present in the Camk2a localizing sequence.

RNAcompete position weight matrices were utilized to identify RBP motifs in the Camk2a UTR (position 3000-3200). Log odds ratios for all 218 tested RBP motifs included in the analysis are reported in descending order.

Table S6. Tile-based measures of RNA localization potential for 229 genes

This table reports the measures computed on all the 150 nt tiles taken from the native 3'UTR of the 229 genes analyzed (at least 10 tiles analyzed, 3'UTR length at least 350 nt). It includes the length of the 3'UTR and the number of sequences tested as well as readouts for mean logFC(neurite/soma) for all tiles or only those with significant enrichment in either compartment, the fraction of tiles showing significant enrichment in either compartment, and the skew of the distribution of logFC(neurite/soma) and directional p-value ("dirpval":

-log10(p-value)*sign(logFC(neurite/som))). It also includes the number of peaks identified in each gene and its position and height (summarized in greater detail in Tables S3 and S4).

Table S7. RBP motifs positively correlated with logFC(neurite/soma) of library sequences

A list of the significant positive correlations between cumulative RBP binding motif scores (based on RNAcompete) and logFC(neurite/soma) as measured for all tiles of native 3'UTRs in CAD cells (Pearson correlation coefficients and the associated p-value and q-value at FDR=0.1).

Table S8. RBP motifs negatively correlated with logFC(neurite/soma) of library sequences

A list of the significant negative correlations between cumulative RBP binding motif scores (based on RNAcompete) and logFC(neurite/soma) as measured for all tiles of native 3'UTRs in CAD cells (Pearson correlation coefficients and the associated p-value and q-value at FDR=0.1).

Table S9. Correlation analysis of RBP motifs and RNA decay rates

A list of the correlations between cumulative RBP binding motif scores (based on RNAcompete) and logFC(4h/0h Actinomycin D) as measured for all tiles of native 3'UTRs in CAD cells (Pearson correlation coefficients and the associated p-value and q-value at FDR=0.1).

Table S10. Enrichment of RBP motifs in neurite enriched tiles vs. all tiles

Analysis of the enrichment of RBP motifs in neurite enriched tiles compared to all library sequences using the enrich_motifs function from the universalmotif R package, reporting the number of motif hits for each of the RBP top 10 n-mers as well as the corresponding p-value and q-value. The reported enriched motifs are selected as the ones having a p-value < 0.001 in Fisher's Exact Test for the counts.

Table S11. Enrichment of RBP motifs in soma enriched tiles vs. all tiles

Analysis of the enrichment of RBP motifs in soma enriched tiles compared to all library sequences using the enrich_motifs function from the universalmotif R package, reporting the number of motif hits for each of the RBP top 10 n-mers as well as the corresponding p-value

and q-value. The reported enriched motifs are selected as the ones having a p-value < 0.001 in Fisher's Exact Test for the counts.

Table S12. Motif enrichment analysis on published neurite and soma transcriptome datasets

Analysis of the enrichment of RBP motifs in neurite or soma localizing transcripts as measured by Zappulo et al. (13) or Middleton et al. (45), compared to all analyzed transcripts. The number of motif hits for each of the RBP top 10 n-mers as well as the corresponding p-value and q-value are reported.

Table S13. Consensus sequences derived from published neurite and soma transcriptome datasets

Consensus sequences identified by the MEME suite's motif discovery tool, enriched in neurite or soma localizing transcripts as measured by Zappulo et al. (13) or Middleton et al. (45) compared to the corresponding control sequences (Methods). 20 motifs and their best possible match are reported per condition/dataset, the first column indicates whether this particular motif was used in the library design (some motifs were excluded as they would result in hard to synthesize homopolymeric stretches).

Table S14. Sequences of a synthetic neurite-enriched sequence (SU1) and four soma-restricted library sequences used in the pull-down experiments

Table S15. Mass spectrometry results (SU1 vs. soma restricted sequences), CAD cells.

Results of the label-free protein quantification experiment. ProteinName lists all the proteins that were identified with the same set of peptides. Multiple isoforms could be listed together in this column. TopProteinName list only the first protein of this list, in alphabetical order.

nrPeptides number of detected peptides. N2A_Ctrl average expression (transformed values) N2A_probe average expression (transformed values). proteinID, log2FC log2 fold change, CI.L, CI.R confidence interval. AveExpr average expression. P.Value (moderated t-test, Limma (72). adj.P.Val adjusted P value, raw expression value columns, transformed expression value columns.

Table S16. Mass spectrometry results (SU1 vs. soma restricted sequences), Neuro-2a cells.

Results of the label-free protein quantification experiment. ProteinName lists all the proteins that were identified with the same set of peptides. Multiple isoforms could be listed together in this column. TopProteinName lists only the first protein of this list, in alphabetical order.

nrPeptides number of detected peptides. N2A_Ctrl average expression (transformed values) N2A_probe average expression (transformed values). proteinID, log2FC log2 fold change, CI.L, CI.R confidence interval. AveExpr average expression. P.Value (moderated t-test, Limma (72). adj.P.Val adjusted P value, raw expression value columns, transformed expression value columns.

Table S17. List of RBPs enriched in the pull-down using the SU1 sequence over the pool of background sequences

This list represents the overlap of all three groups (identified in CAD, identified in Neuro-2a and mouse RBPs) in the Venn diagram in Figure 6D.

Table S18. RBP motifs present in the SU1 sequence (RBPmap).

RBPmap output. Protein: RBP identity, motif, utilized k-mer, Z-score measures the deviation of the site's weighted rank score from the mean score calculated using the genome-specific background, p value represents the probability of obtaining a specific z-score considering a normal one-tailed distribution (66).

Table S19. Sequence of the smFISH probes used to detect the gfp coding sequence.

Figure S1

A

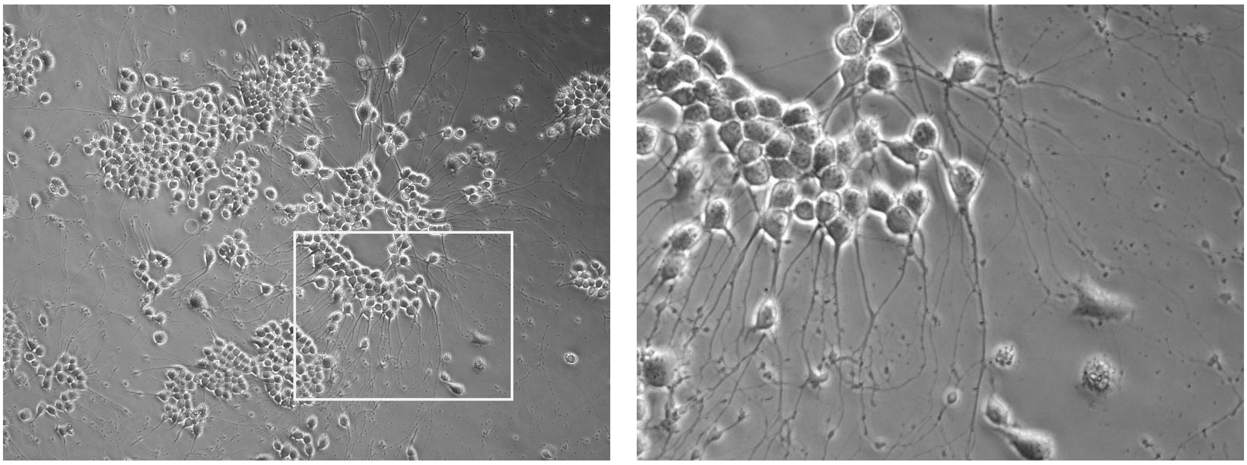

B

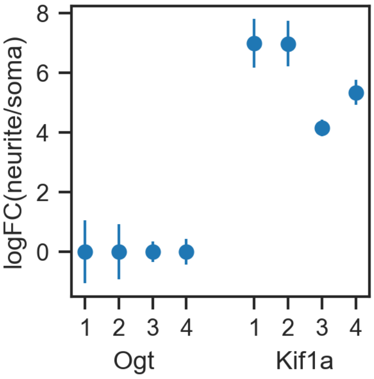

Figure S2

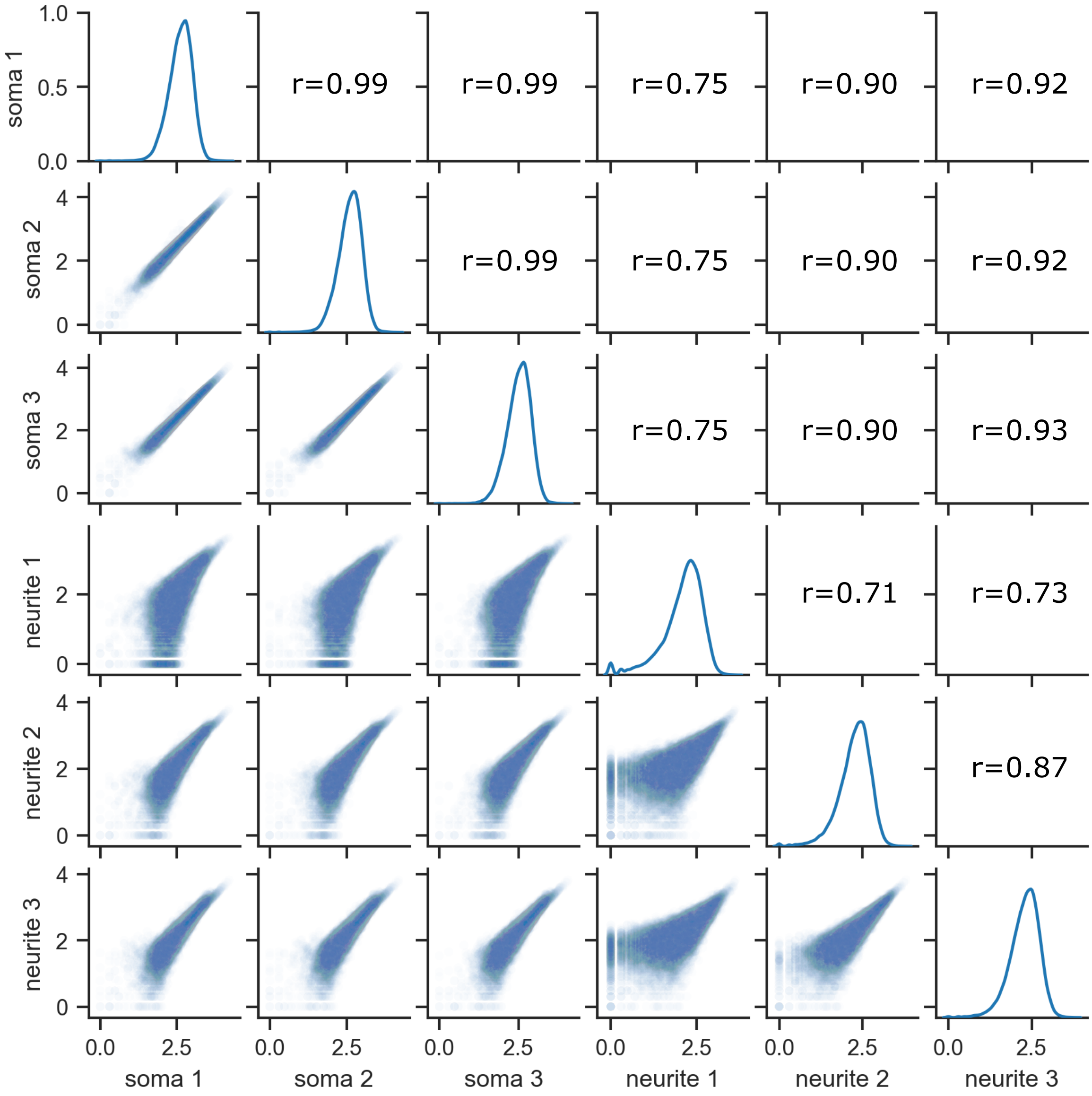

soma 1
soma 2
soma 3
neurite 1
neurite 2
neurite 3
r=0.99
r=0.99
r=0.75
r=0.90
r=0.92
r=0.99
r=0.75
r=0.90
r=0.92
r=0.75
r=0.90
r=0.93
r=0.71
r=0.73
r=0.87
soma 1
soma 2
soma 3
neurite 1
neurite 2
neurite 3

Figure S3

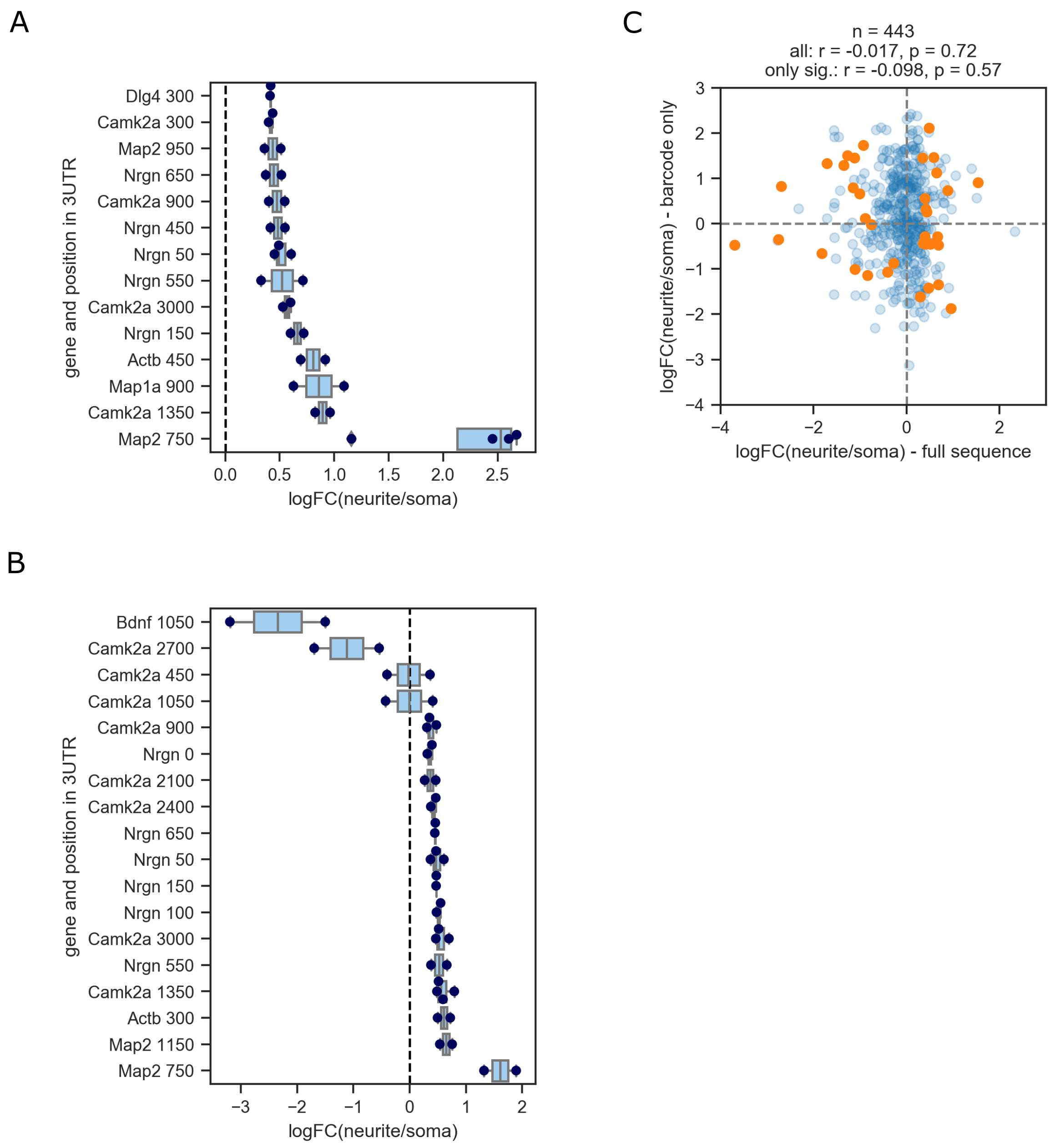
A
Dlg4 300
Camk2a 300
Map2 950
Nrgn 650
Camk2a 900
Nrgn 450
Nrgn 50
Nrgn 550
Camk2a 3000
Nrgn 150
Actb 450
Map1a 900
Camk2a 1350
Map2 750
gene and position in 3UTR
0.0 0.5 1.0 1.5 2.0 2.5
logFC(neurite/soma)
B
Bdnf 1050
Camk2a 2700
Camk2a 450
Camk2a 1050
Camk2a 900
Nrgn 0
Camk2a 2100
Camk2a 2400
Nrgn 650
Nrgn 50
Nrgn 150
Nrgn 100
Camk2a 3000
Nrgn 550
Camk2a 1350
Actb 300
Map2 1150
Map2 750
gene and position in 3UTR
−3 −2 −1 0 1 2
logFC(neurite/soma)
C
n = 443
all: r = -0.017, p = 0.72
only sig.: r = -0.098, p = 0.57
logFC(neurite/soma) - barcode only
3 2 1 0 −1 −2 −3 −4
−4 −2 0 2
logFC(neurite/soma) - full sequence

Figure S4

A

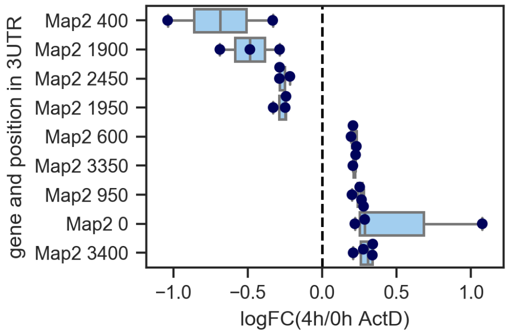
Map2 400
Map2 1900
Map2 2450
Map2 1950
Map2 600
Map2 3350
Map2 950
Map2 0
Map2 3400
gene and position in 3UTR
−1.0
−0.5
0.0
0.5
1.0
logFC(4h/0h ActD)

B

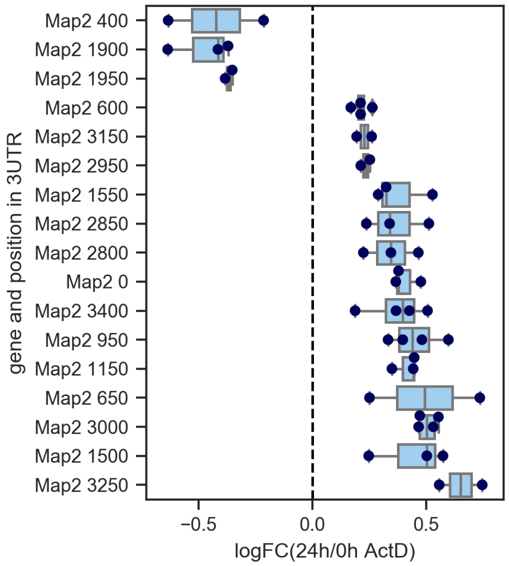
Map2 400
Map2 1900
Map2 1950
Map2 600
Map2 3150
Map2 2950
Map2 1550
Map2 2850
Map2 2800
Map2 0
Map2 3400
Map2 950
Map2 1150
Map2 650
Map2 3000
Map2 1500
Map2 3250
gene and position in 3UTR
−0.5
0.0
0.5
logFC(24h/0h ActD)

Figure S5

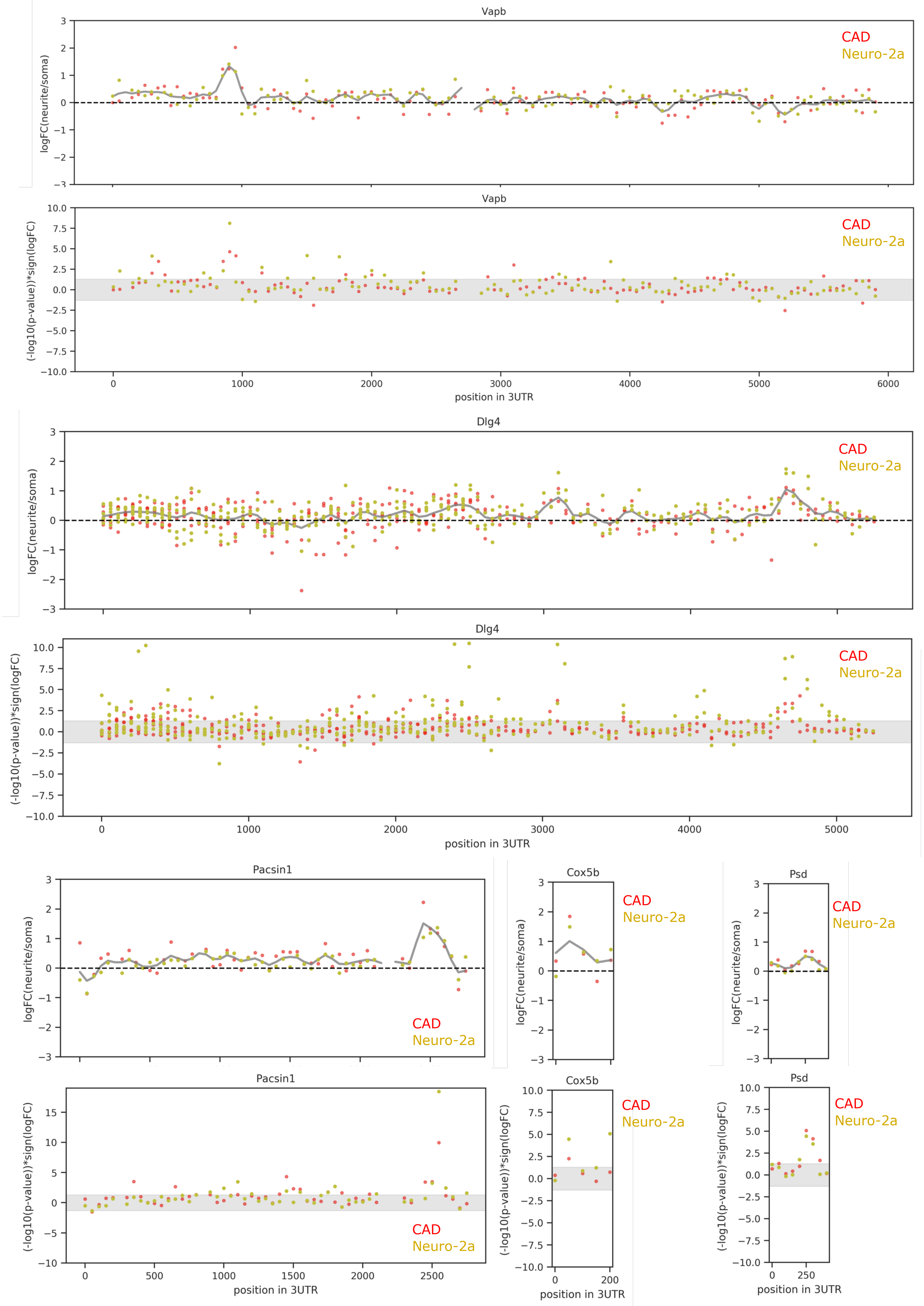
Vapb
logFC(neurite/soma)
(-log10(p-value))*sign(logFC)
position in 3UTR
CAD
Neuro-2a
Dlg4
Pacsin1
Cox5b
Psd

Figure S6

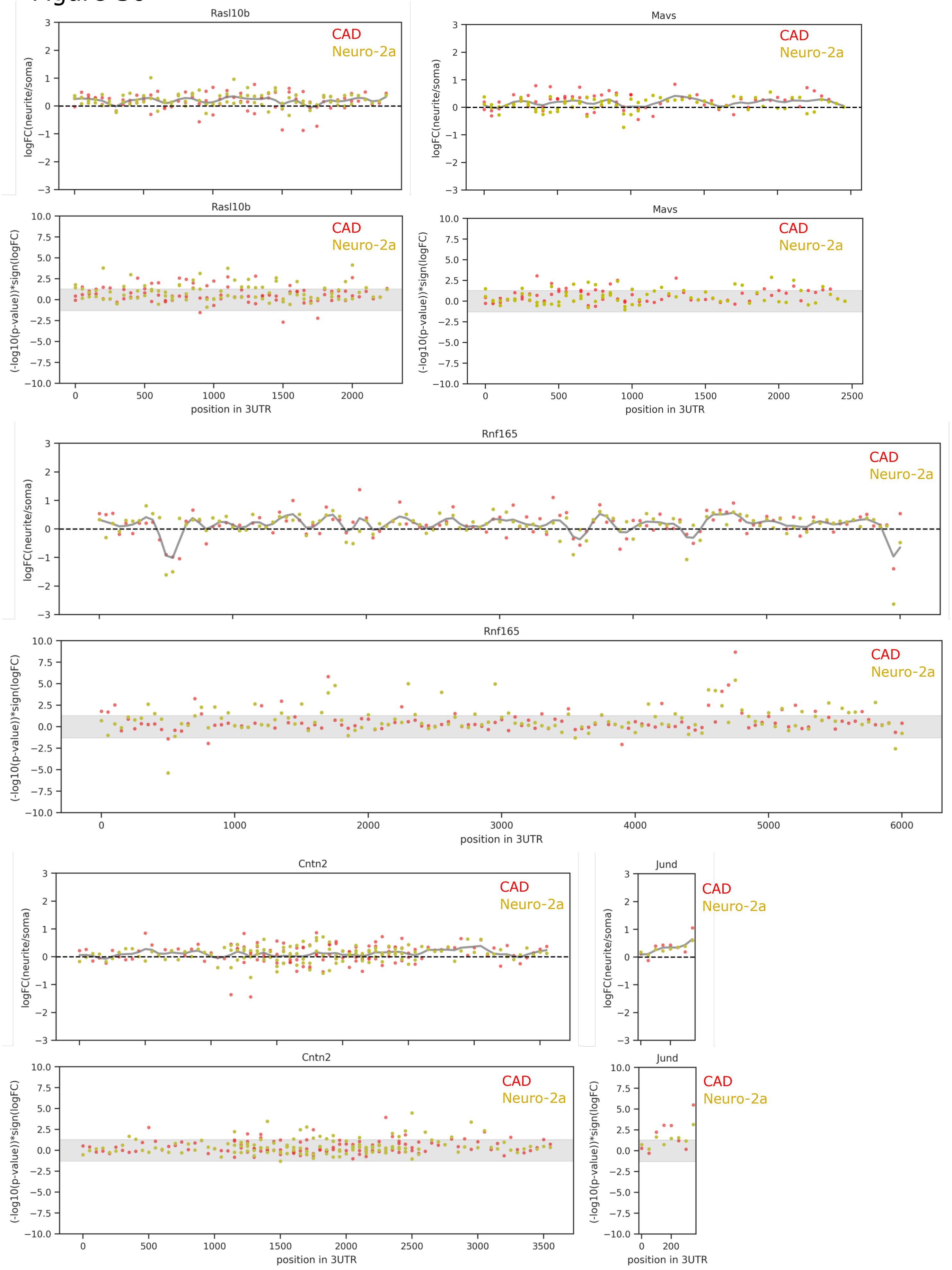
Rasl10b
Mavs
Rnf165
Cntn2
Jund
CAD
Neuro-2a
logFC(neurite/soma)
(-log10(p-value))*sign(logFC)
position in 3UTR

Figure S7

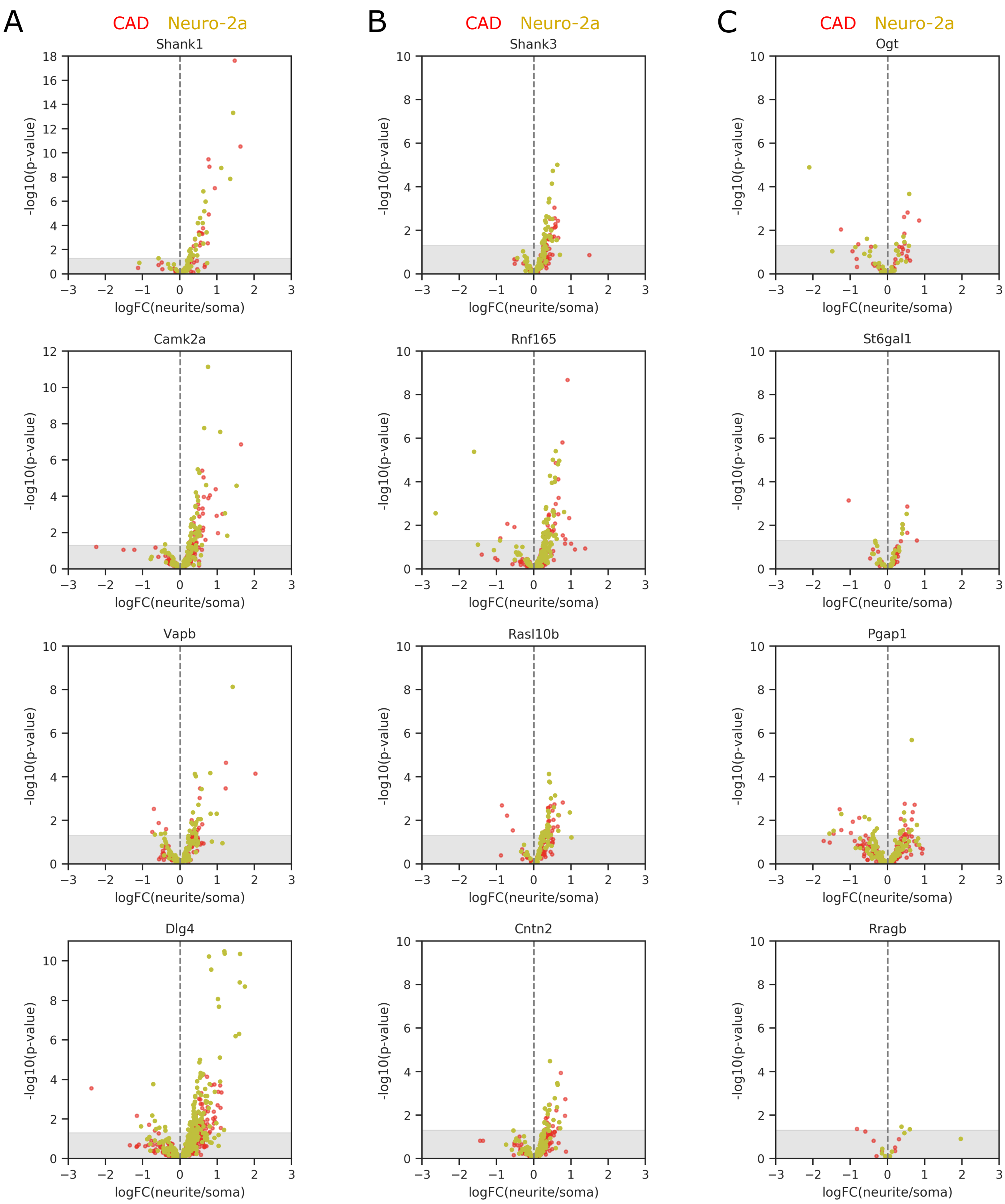
A
B
C
CAD
Neuro-2a
Shank1
Camk2a
Vapb
Dlg4
Shank3
Rnf165
Rasl10b
Cntn2
Ogt
St6gal1
Pgap1
Rragb
-log10(p-value)
logFC(neurite/soma)

Figure S8

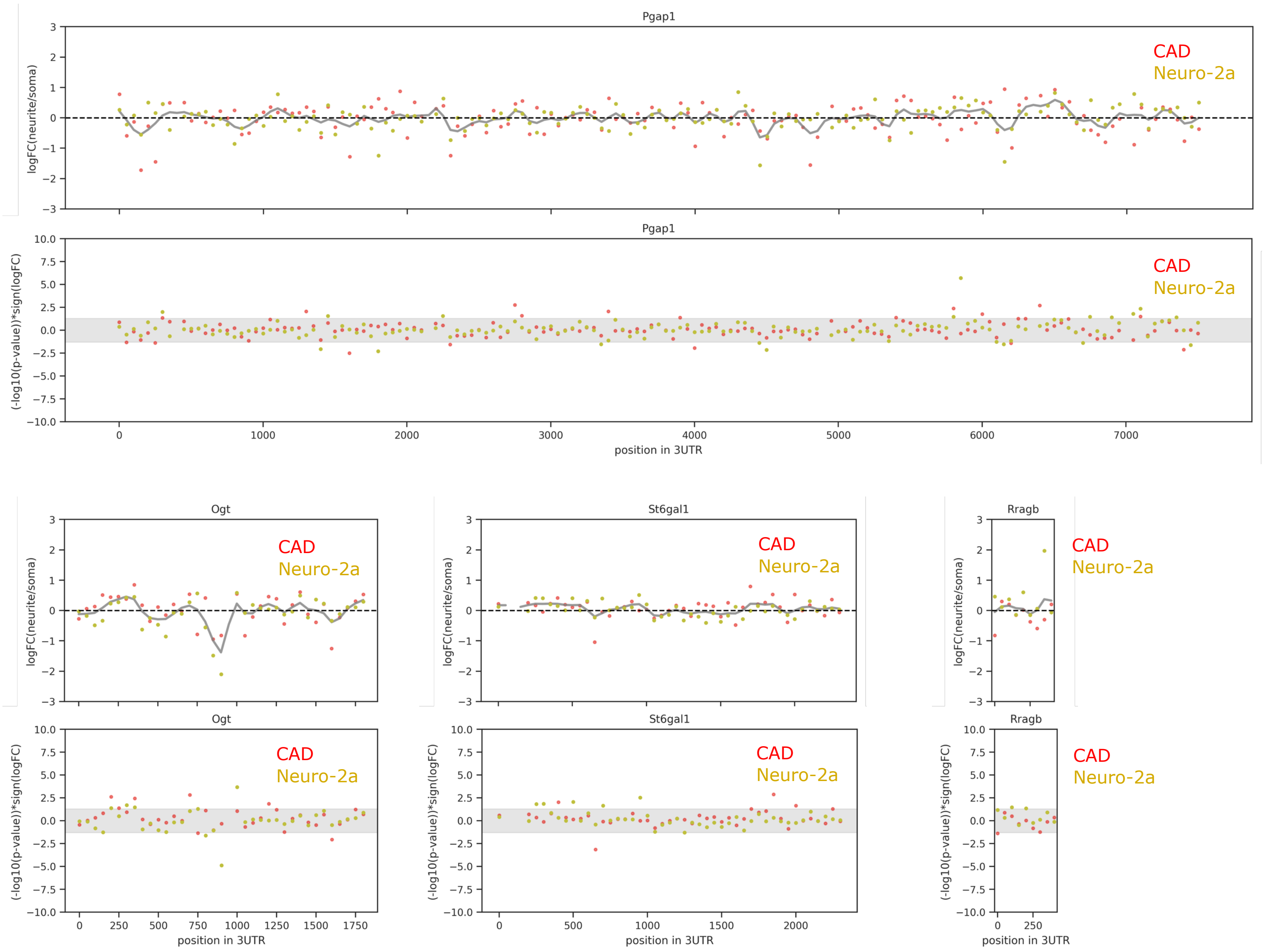
Pgap1
logFC(neurite/soma)
CAD
Neuro-2a
Pgap1
(-log10(p-value))*sign(logFC)
position in 3UTR
Ogt
St6gal1
Rragb
position in 3UTR
position in 3UTR
position in 3UTR

Figure S9

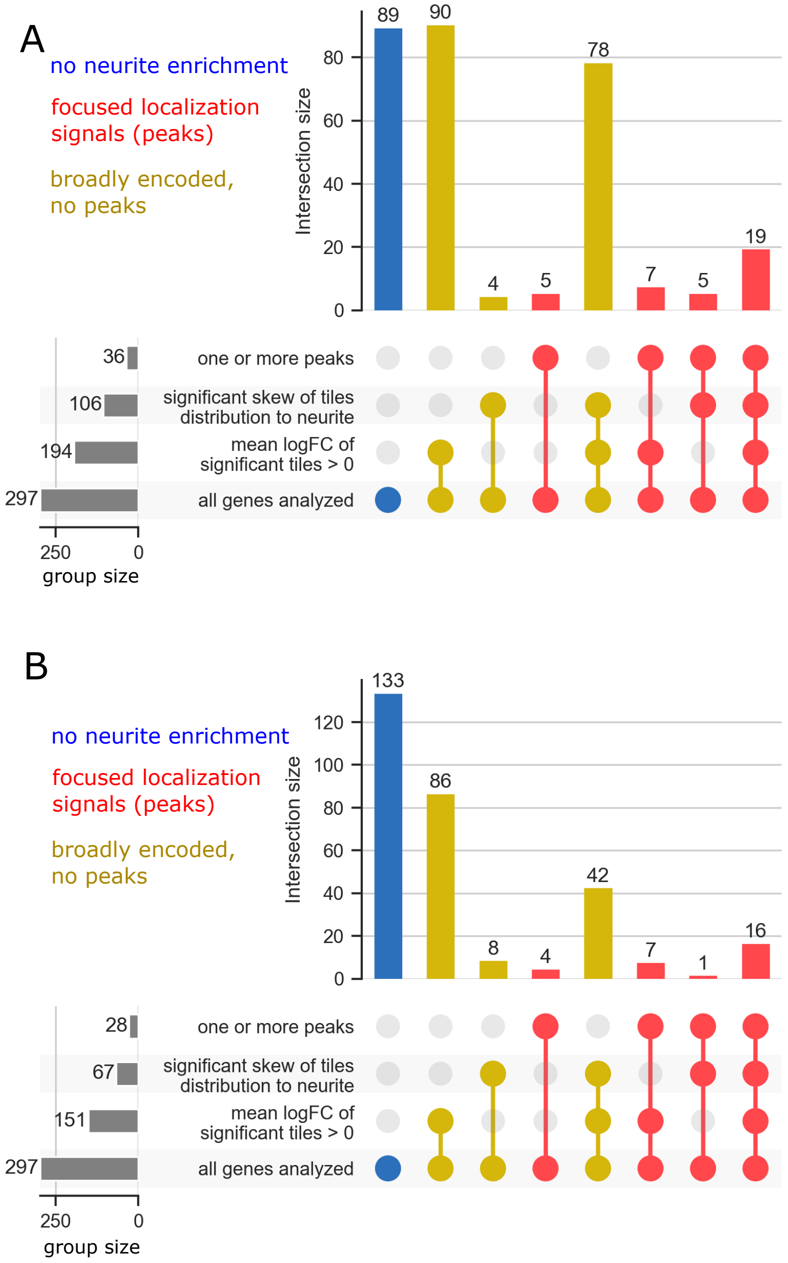

A
no neurite enrichment
focused localization signals (peaks)
broadly encoded, no peaks
Intersection size
89
90
4
5
78
7
5
19
one or more peaks
significant skew of tiles distribution to neurite
mean logFC of significant tiles > 0
all genes analyzed
36
106
194
297
250
0
group size
B
no neurite enrichment
focused localization signals (peaks)
broadly encoded, no peaks
Intersection size
133
86
8
4
42
7
1
16
one or more peaks
significant skew of tiles distribution to neurite
mean logFC of significant tiles > 0
all genes analyzed
28
67
151
297
250
0
group size

Figure 10

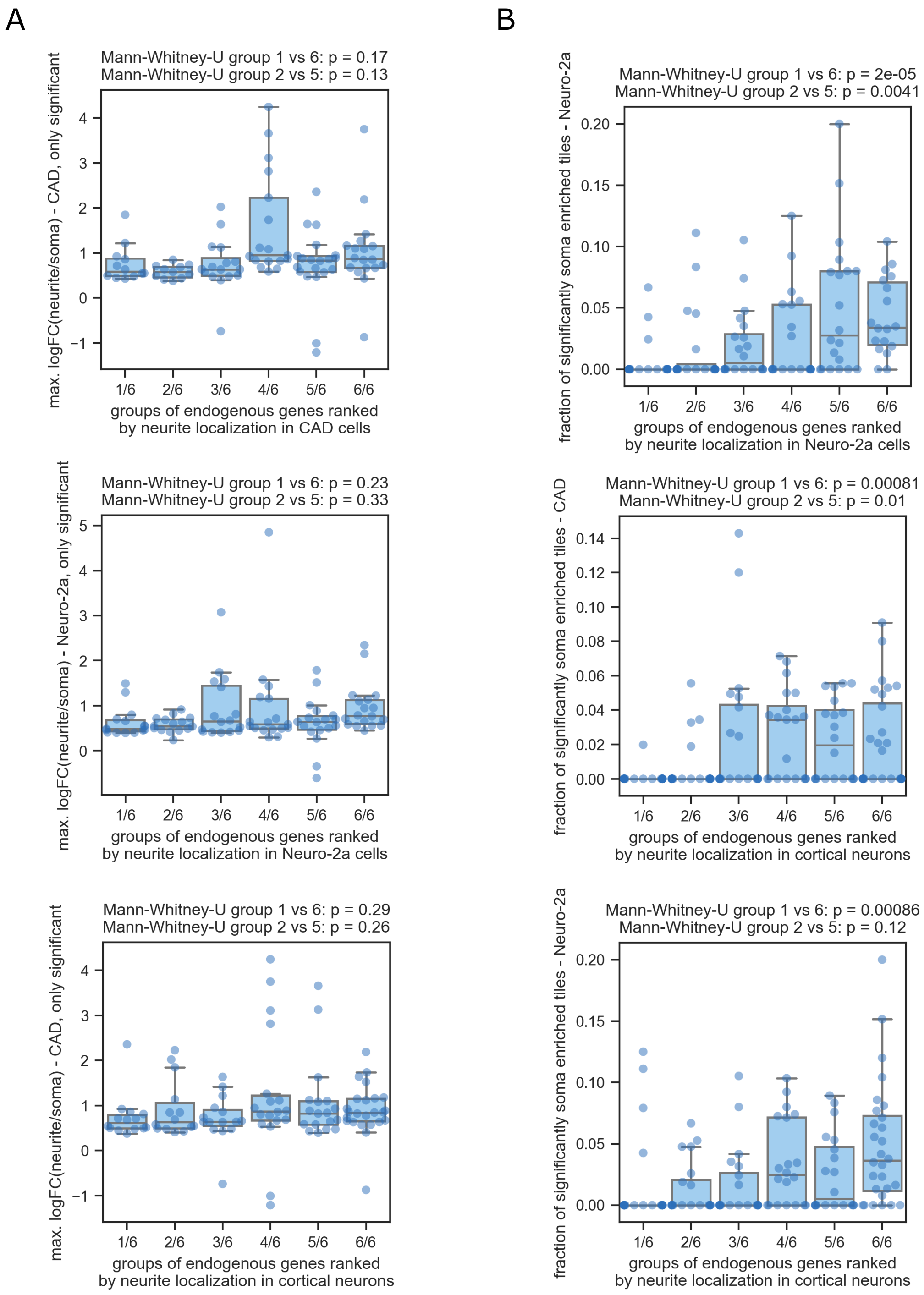
A
B
Mann-Whitney-U group 1 vs 6: p = 0.17
Mann-Whitney-U group 2 vs 5: p = 0.13
max. logFC(neurite/soma) - CAD, only significant
groups of endogenous genes ranked
by neurite localization in CAD cells
Mann-Whitney-U group 1 vs 6: p = 2e-05
Mann-Whitney-U group 2 vs 5: p = 0.0041
fraction of significantly soma enriched tiles - Neuro-2a
groups of endogenous genes ranked
by neurite localization in Neuro-2a cells
Mann-Whitney-U group 1 vs 6: p = 0.23
Mann-Whitney-U group 2 vs 5: p = 0.33
max. logFC(neurite/soma) - Neuro-2a, only significant
groups of endogenous genes ranked
by neurite localization in Neuro-2a cells
Mann-Whitney-U group 1 vs 6: p = 0.00081
Mann-Whitney-U group 2 vs 5: p = 0.01
fraction of significantly soma enriched tiles - CAD
groups of endogenous genes ranked
by neurite localization in cortical neurons
Mann-Whitney-U group 1 vs 6: p = 0.29
Mann-Whitney-U group 2 vs 5: p = 0.26
max. logFC(neurite/soma) - CAD, only significant
groups of endogenous genes ranked
by neurite localization in cortical neurons
Mann-Whitney-U group 1 vs 6: p = 0.00086
Mann-Whitney-U group 2 vs 5: p = 0.12
fraction of significantly soma enriched tiles - Neuro-2a
groups of endogenous genes ranked
by neurite localization in cortical neurons
1/6
2/6
3/6
4/6
5/6
6/6

Figure S11

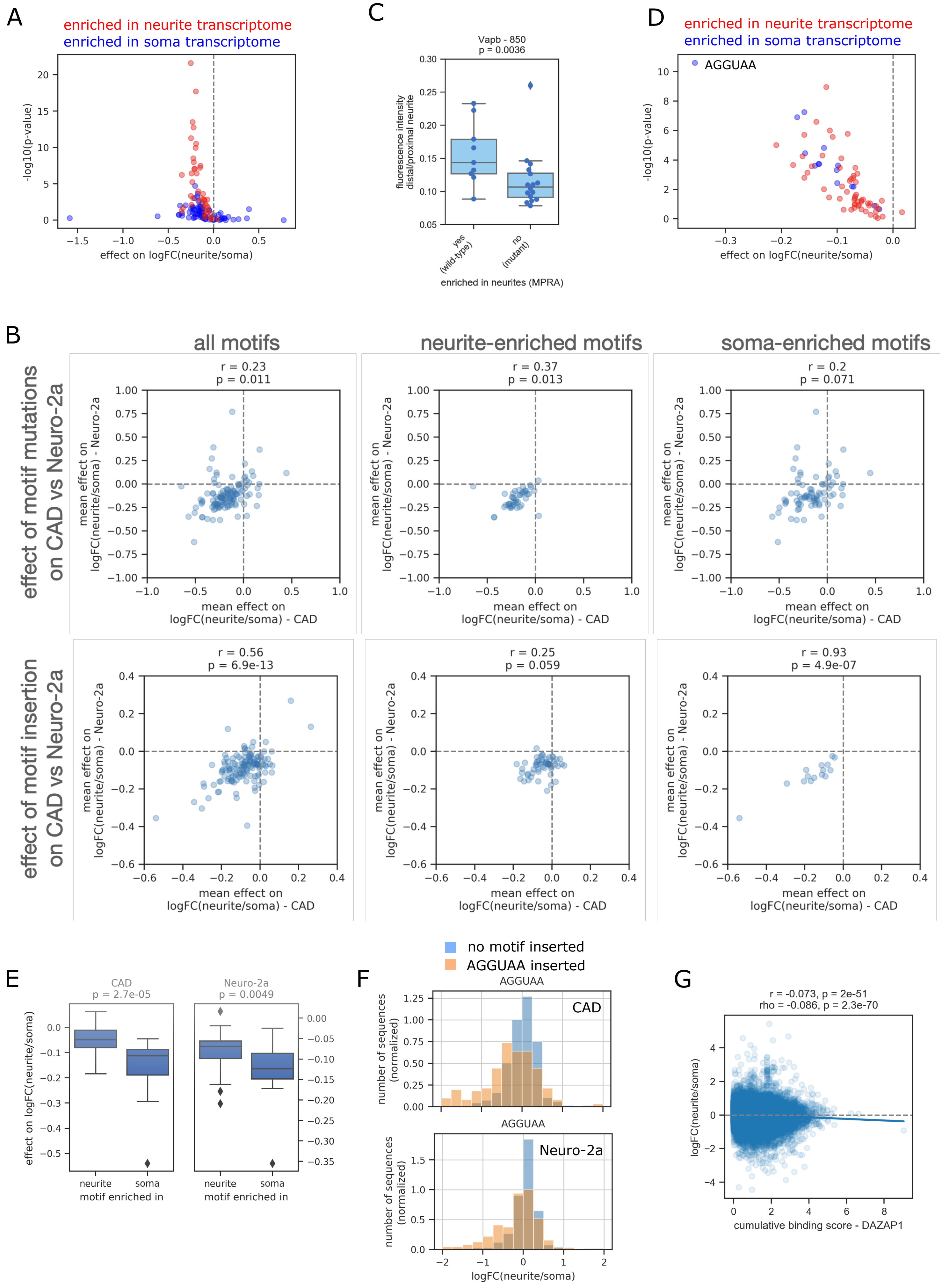

A
enriched in neurite transcriptome
enriched in soma transcriptome
-log10(p-value)
effect on logFC(neurite/soma)
C
Vapb - 850
p = 0.0036
fluorescence intensity distal/proximal neurite
yes (wild-type)
no (mutant)
enriched in neurites (MPRA)
D
enriched in neurite transcriptome
enriched in soma transcriptome
AGGUAA
-log10(p-value)
effect on logFC(neurite/soma)
B
all motifs
neurite-enriched motifs
soma-enriched motifs
effect of motif mutations on CAD vs Neuro-2a
r = 0.23
p = 0.011
r = 0.37
p = 0.013
r = 0.2
p = 0.071
effect of motif insertion on CAD vs Neuro-2a
r = 0.56
p = 6.9e-13
r = 0.25
p = 0.059
r = 0.93
p = 4.9e-07
mean effect on logFC(neurite/soma) - Neuro-2a
mean effect on logFC(neurite/soma) - CAD
E
CAD
p = 2.7e-05
Neuro-2a
p = 0.0049
effect on logFC(neurite/soma)
neurite
soma
motif enriched in
F
no motif inserted
AGGUAA inserted
AGGUAA
CAD
Neuro-2a
number of sequences (normalized)
logFC(neurite/soma)
G
r = -0.073, p = 2e-51
rho = -0.086, p = 2.3e-70
logFC(neurite/soma)
cumulative binding score - DAZAP1

Figure S12

A

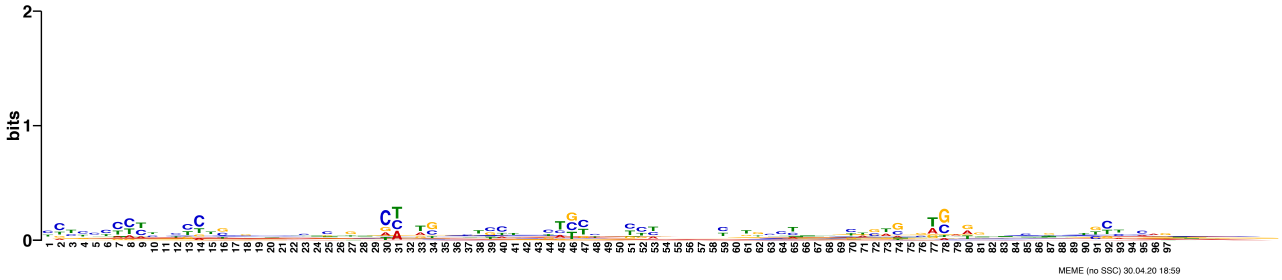

B

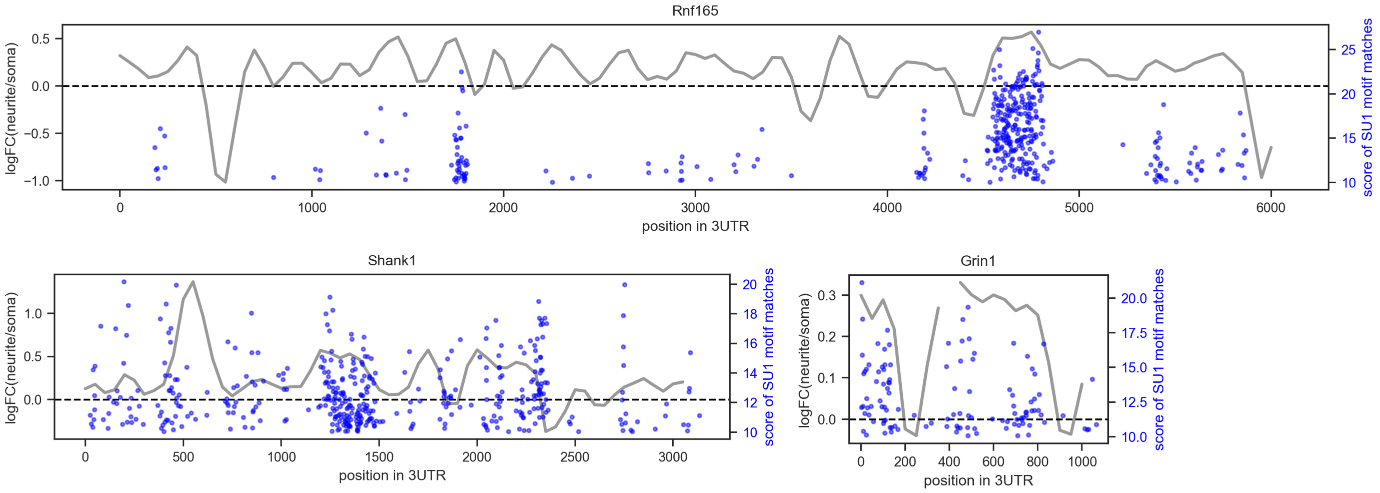

Figure S13

A

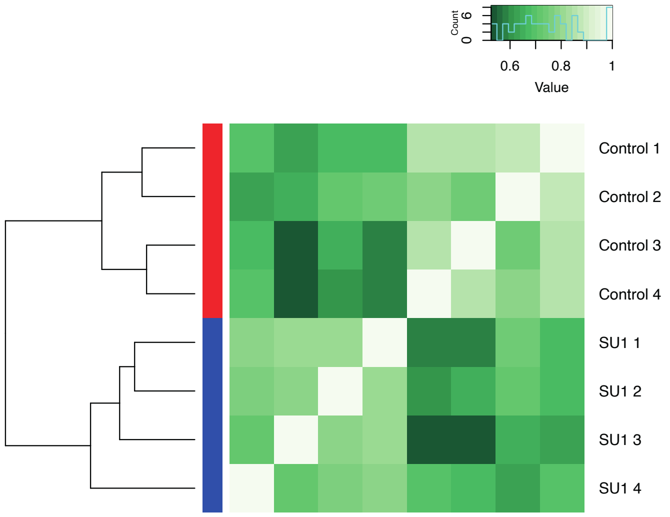

B

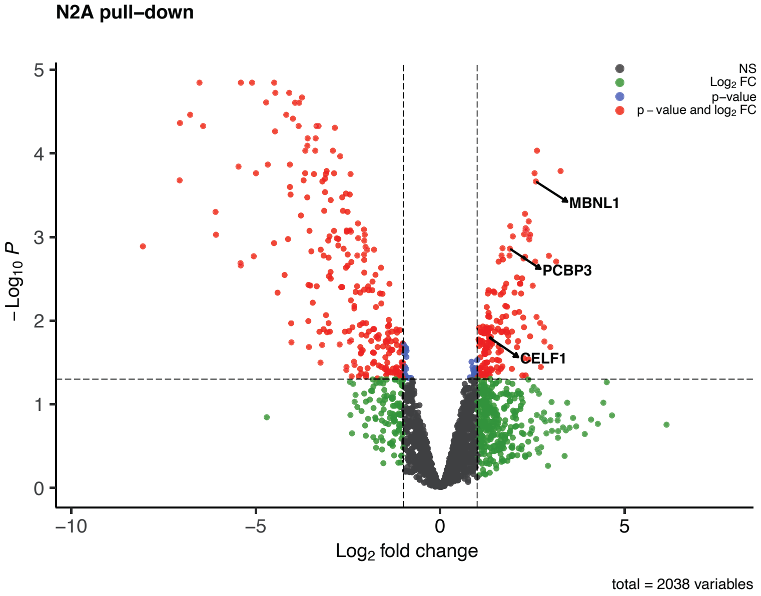

Figure S14

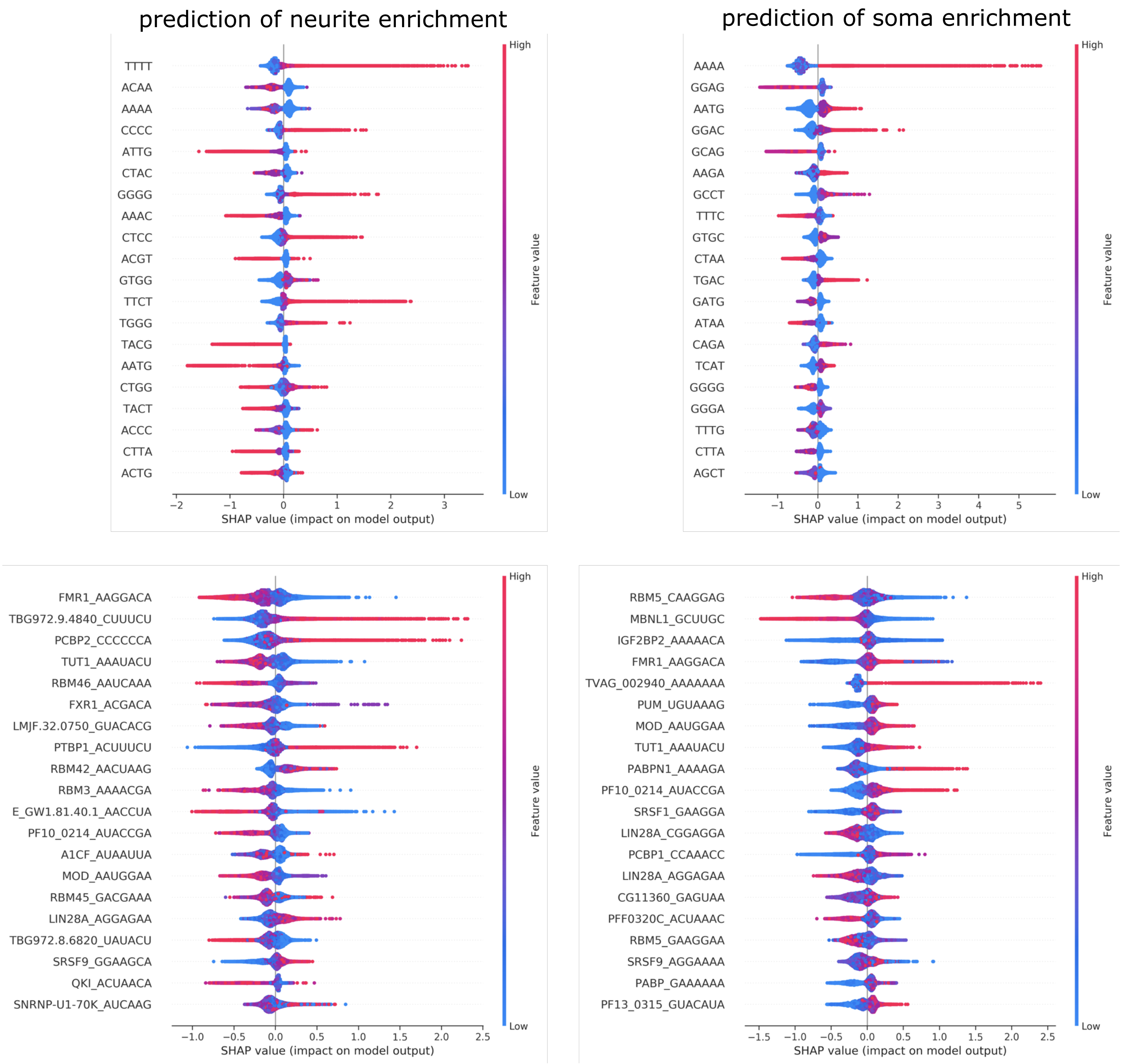
prediction of neurite enrichment
prediction of soma enrichment
TTTT
ACAA
AAAA
CCCC
ATTG
CTAC
GGGG
AAAC
CTCC
ACGT
GTGG
TTCT
TGGG
TACG
AATG
CTGG
TACT
ACCC
CTTA
ACTG
SHAP value (impact on model output)
Feature value
High
Low
AAAA
GGAG
AATG
GGAC
GCAG
AAGA
GCCT
TTTC
GTGC
CTAA
TGAC
GATG
ATAA
CAGA
TCAT
GGGG
GGGA
TTTG
CTTA
AGCT
SHAP value (impact on model output)
Feature value
High
Low
FMR1_AAGGACA
TBG972.9.4840_CUUUCU
PCBP2_CCCCCCA
TUT1_AAAUACU
RBM46_AAUCAAA
FXR1_ACGACA
LMJF.32.0750_GUACACG
PTBP1_ACUUUCU
RBM42_AACUAAG
RBM3_AAAACGA
E_GW1.81.40.1_AACCUA
PF10_0214_AUACCGA
A1CF_AUAAUUA
MOD_AAUGGAA
RBM45_GACGAAA
LIN28A_AGGAGAA
TBG972.8.6820_UAUACU
SRSF9_GGAAGCA
QKI_ACUAACA
SNRNP-U1-70K_AUCAAG
SHAP value (impact on model output)
Feature value
High
Low
RBM5_CAAGGAG
MBNL1_GCUUGC
IGF2BP2_AAAAACA
FMR1_AAGGACA
TVAG_002940_AAAAAAA
PUM_UGUAAAG
MOD_AAUGGAA
TUT1_AAAUACU
PABPN1_AAAAGA
PF10_0214_AUACCGA
SRSF1_GAAGGA
LIN28A_CGGAGGA
PCBP1_CCAAACC
LIN28A_AGGAGAA
CG11360_GAGUAA
PFF0320C_ACUAAAC
RBM5_GAAGGAA
SRSF9_AGGAAAA
PABP_GAAAAAA
PF13_0315_GUACAUA
SHAP value (impact on model output)
Feature value
High
Low

Figure S15

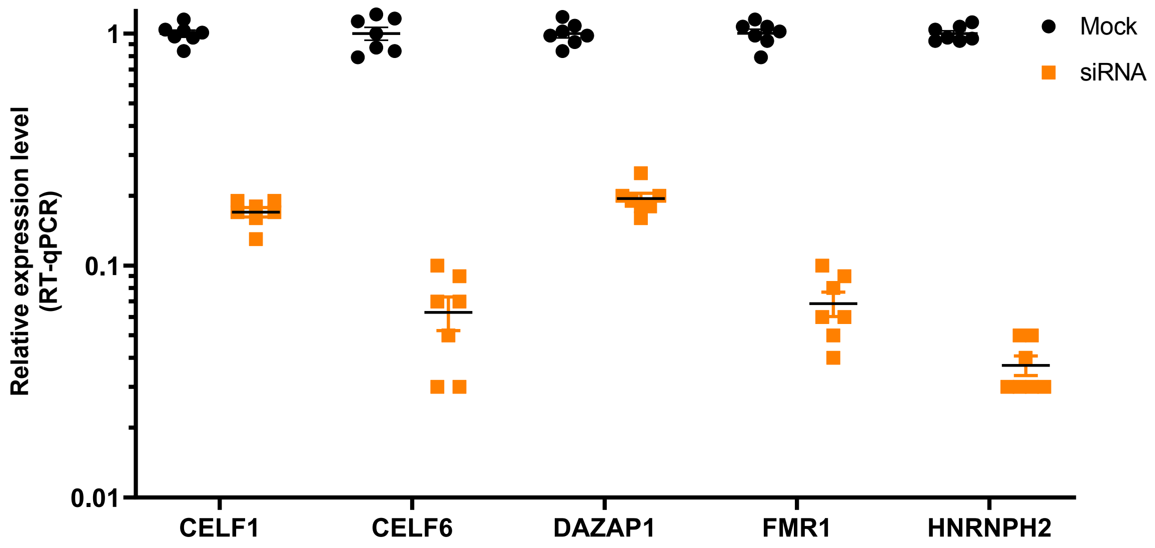

Relative expression level
(RT-qPCR)
1
0.1
0.01
CELF1
CELF6
DAZAP1
FMR1
HNRNPH2
Mock
siRNA
